# Enzymatically Bioactive Nucleus Pulposus Matrix Hydrogel Microspheres for Exogenous Stem Cells Therapy and Endogenous Repair Strategy to Achieve Disc Regeneration

**DOI:** 10.1002/advs.202304761

**Published:** 2023-12-25

**Authors:** Yizhong Peng, Xuanzuo Chen, Qimin Zhang, Sheng Liu, Wei Wu, Kanglu Li, Hui Lin, Xiangcheng Qing, Yan Xiao, BaiChuan Wang, Daping Quan, Shiqing Feng, Zilong Rao, Ying Bai, Zengwu Shao

**Affiliations:** ^1^ Department of Orthopedics Union Hospital Tongji Medical College Huazhong University of Science and Technology Wuhan 430022 China; ^2^ Department of Radiology Wuhan Third Hospital Tongren Hospital of Wuhan University 241 Pengliuyang Road Wuhan Hubei 430063 China; ^3^ Department of Radiology Union Hospital Tongji Medical College Huazhong University of Science and Technology Wuhan 430022 China; ^4^ School of Materials Science and Engineering Sun Yat‐sen University Guangzhou 510127 China; ^5^ The Second Hospital of Shandong University Cheeloo College of Medicine Shandong University Jinan Shandong 250033 P. R. China; ^6^ Department of Orthopaedics Tianjin Medical University General Hospital Tianjin Medica University International Science and Technology Cooperation Base of Spinal Cord Injury Tianjin Key Laboratory of Spine and Spinal Cord Tianjin 300052 P. R. China; ^7^ Department of Orthopaedics Qilu Hospital of Shandong University Shandong University Centre for Orthopaedics Advanced Medical Research Institute Cheeloo College of Medicine Shandong University Jinan Shandong 250012 P. R. China

**Keywords:** autophagy, intervertebral disc degeneration, lactate, microfluidic system, nanozyme, TGFB2‐OT1

## Abstract

Exogenous stem cell therapy and endogenous repair has shown great potential in intervertebral disc regeneration. However, limited nutrients and accumulation of lactate largely impair the survival and regenerative capacity of implanted stem cells and endogenous nucleus pulposus cells (NPCs). Herein, an injectable hydrogel microsphere (LMGDNPs) have been developed by immersing lactate oxidase (LOX)‐manganese dioxide (MnO_2_) nanozyme (LM) into glucose‐enriched decellularized nucleus pulposus hydrogel microspheres (GDNPs) through a microfluidic system. LMGDNPs showed a delayed release profile of LOX and satisfactory enzymatic capacity in consuming lactate. Mesenchymal stem cells (MSCs) plated on LMGDNPs exhibited better cell viability than cells on GelMA and decellularized nucleus pulposus microspheres (DNP) and showed a obviously increased NPCs phenotype. LMGDNPs prevented MSCs and NPCs death and promoted extracellular matrix synthesis by exhausting lactate. It is determined that LMGDNPs promoted NPCs autophagy by activating transforming growth factor β2 overlapping transcript 1 (TGFB2‐OT1), relying on the nanozyme. MSCs‐loaded LMGDNPs largely preserved disc hydration and alleviated matrix degradation in vivo. Summarily, LMGDNPs promoted cell survival and matrix regeneration by providing a nutrient supply, exhausting lactate, and activating autophagy via TGFB2‐OT1 and its downstream pathway and may serve as an ideal delivery system for exogenous stem cell therapy and endogenous repair.

## Graphical abstract

LOX‐MnO_2_ nanozyme‐loaded glucose‐rich nucleus pulposus matrix hydrogel‐based microspheres (LMGDNPs), as carriers of exogenous stem cells, induce directional differentiation of stem cells into nucleus pulposus cells. LMGDNPs reduce cell death and extracellular matrix breakdown by consuming lactate. LM released from LMGDNPs promotes the formation of autophagosomes through the TGFB2‐OT1/miRNAs/ATGs/NAT8L/CERS1 pathway and consumes lactate to inhibit the blocking effect of lactate on autophagy flow, thereby activating autophagy, promoting cell survival and matrix regeneration.

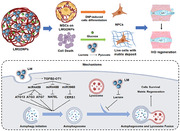

## 1 Introduction

Low back pain (LBP) is the leading cause of health conditions that contribute to the need for rehabilitation services worldwide.^[^ ^1^ ^]^ Intervertebral disc (IVD) degeneration (IDD), a primary pathological contributor to LBP, is expected to emerge on a larger scale with global aging, imposing tremendous economic and social burdens.^[^ ^2^, ^3^ ^]^ Based on clinical data, conventional treatments for IDD include physiotherapy, nonsteroidal anti‐inflammatory drugs, epidural injection, surgical decompression, fusion, and disk replacement. However, the adverse effects following prolonged drug administration, the incidence of reherniation, and adjacent disc degeneration after surgery impair the therapeutic effects of these traditional therapies.^[^ ^4^, ^5^, ^6^ ^]^

Exogenous stem cell‐based therapies combine tissue engineering science and biomaterials technologies and serve as the fundamental pillars of regenerative medicine.^[^ ^7^ ^]^ Due to their self‐renewal capacity, ease of isolation, multilineage differentiation potential, and immunomodulatory properties, mesenchymal stem cells (MSCs) implantation is an attractive option for IDD therapies.^[^ ^8^ ^]^ Apart from exogenous stem cells, endogenous repair involves local tissue repair that relies on the naturally available endogenous cells in situ.^[^ ^9^ ^]^ The residual viable cells are capable of reproducing extracellular matrix (ECM). Maintaining the viability and functionality of endogenous cells after tissue injury has been identified as a major strategy for alleviating IDD.^[^ ^10^ ^]^

Due to the avascular environment, nutrients in the IVD are supplied by vessels at its margins, restricting the transport of nutrients into the center of the IVD.^[^ ^11^ ^]^ Therefore, discs can only support a limited number of cells as nutrient concentrations decrease with distance from the vessels. When IDD occurs, occlusion of marrow spaces, calcified cartilaginous endplates and atherosclerosis of lumbar arteries all lead to decreased nutrient supply.^[^ ^12^, ^13^ ^]^ As the cell number increases after MSC implantation, the nutrient demand also increases, and further cell death ensues with implanted MSCs competing with the remaining viable resident disc cells for available nutrients.^[^ ^13^ ^]^ Although they can effectively modulate inflammation and stimulate matrix production, MSCs have to be alive and supplied with enough energy, especially glucose, to maintain their activities.^[^ ^14^ ^]^ Therefore, the imbalance between nutrient demand and supply after MSC implantation, especially in cases of IDD, impairs the efficiency of both stem cell therapy and endogenous repair.

The hypoxic microenvironment in IVD determines that anaerobic glycolysis provides the main source of energy in nucleus pulposus cells (NPCs), leading to the accumulation of the metabolic products of glycolysis in discs. Lactic acid, the main metabolic product of glycolysis, has been reported to be higher in degenerative disc tissues than in blood and other tissues,^[^ ^15^, ^16^ ^]^ which was detrimental to the survival and activity of both MSCs and endogenous disc cells.^[^ ^16^, ^17^ ^]^ Thus, it is necessary to modify the energy metabolism imbalance of IVD and eliminate the adverse effects of metabolites to improve the energy metabolism environment and maintain cells survival and functionality.

Lactate oxidase enzyme (LOX) enzymatically catalyzes lactate oxidation to generate pyruvate and hydrogen peroxide (H_2_O_2_), whereas H_2_O_2_ is a critical component of reactive oxygen species that induces oxidative stress and causes cell death in IDD.^[^ ^18^, ^19^ ^]^ Shen et al. designed a manganese dioxide (MnO_2_)‐based nanozyme.^[^ ^20^ ^]^ Using MnO_2_ to catalyze the decomposition of H_2_O_2_ into oxygen and water, the system metabolized the H_2_O_2_ generated from the consumption of lactic acid, and provided additional O_2_ to facilitate further consumption of lactic acid. However, the synthesis of manganese dioxide (MnO_2_)‐based nanozymes in this report was relatively complicated, requiring four steps and additional heat to reach the reaction temperature of 60°C, which was not conducive to large‐scale industrial production and clinical applications.^[^ ^20^ ^]^ Therefore, we improved the synthesis method of LOX‐MnO_2_ nanozymes, reduced the reaction to two steps, and controlled the reaction temperature to ≈25°C.

Due to their small size, easy diffusion, and easy removal by cells, nanozymes are not conducive to local long‐term enrichment.^[^ ^21^ ^]^ Thus, a delivery system is required to maintain its local activity. Decellularized extracellular matrix (dECM) has drawn increasing attention in regenerative medicine in recent decades owing to its high biocompatibility and suitability as a bioscaffold for tissue regeneration.^[^ ^22^ ^]^ We previously prepared a dECM hydrogel derived from porcine sciatic nerves to fabricate microspheres using a microfluidic system, and the microspheres showed good cytocompatibility and sustained release of small molecules.^[^ ^23^ ^]^ We have previously reported that the dECM hydrogel derived from the nucleus pulposus (NP) matrix effectively promoted the tissue‐specific differentiation of stem cells into NPCs.^[^ ^24^ ^]^ Therefore, we prepared microspheres based on NP matrix hydrogel as a delivery system for stem cells and nanozymes.

This study will utilize microfluidic technology to prepare glucose‐rich NP matrix hydrogel‐based microspheres (GDNPs) to provide nutrient supply and pro‐differentiation clues for MSCs. In addition, a simple synthetic LOX‐MnO_2_ nanozyme (LM) was constructed and mounted on GDNPs to obtain the LOX‐MnO_2_ nanozyme‐loaded glucose‐rich nucleus pulposus matrix hydrogel‐based microspheres (LMGDNPs) that remove excessive lactic acid and reduce the damage of metabolic byproducts to cells. The system is simple to prepare, convenient for storage, injectable, easy to produce on a large scale, and has potential for clinical application. The system is supposed to modulate the nutrient imbalance and harsh microenvironment after stem cells are implanted into discs, which will improve the efficiency of stem cell therapy and endogenous repair for IDD.

## 2 Results

### 2.1 Characteristics of LOX‐MnO_2_ Nanozyme

**Figure 1:**
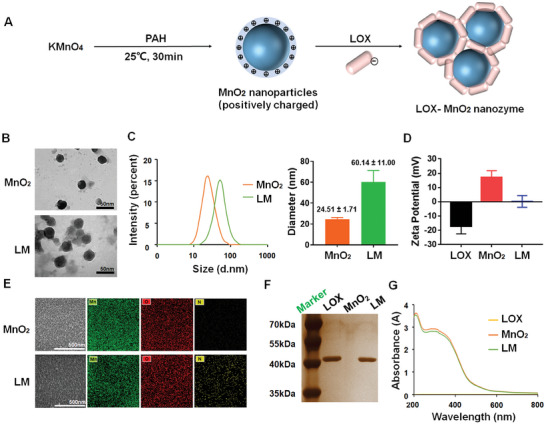
Fabrication and characteristics of LOX‐MnO_2_ nanozyme. A) Schematic overview of the construction of LM. B) TEM images of PAH‐coated MnO_2_ nanoparticles and LM. C) Size distribution of nanoparticles. D) Effect of coating PAH‐MnO_2_ nanoparticles with LOX on zeta potential. Data are presented as the mean ± SD, *n* = 3. E) SEM images and element analysis of nanoparticles. F) Sliver staining of SDS‒PAGE for protein analysis of LOX, PAH‐MnO_2_ nanoparticles, and LM. G) UV–vis absorption spectra of materials and LM. LOX, lactate oxidase. MnO_2_, manganese dioxide. PAH, poly(allylamine hydrochloride). LM, lactate oxidase‐manganese dioxide nanozymes.

A one‐step method was employed to reduce manganese permanganate (KMnO_4_) to MnO_2_ nanoparticles using the cationic polyelectrolyte PAH, generating a protective layer to stabilize MnO_2_ nanoparticles due to electrostatic repulsion. LOX with negative charges was further bonded to the PAH‐coated MnO_2_ nanoparticles through electrostatic interactions, generating the LOX‐MnO_2_ nanozyme (LM) (**Figure** **1**A). PAH‐coated MnO_2_ nanoparticles presented as stable colloidal dispersions, while LOX loading changed the morphological structure and enlarged the average diameter (Figure 1B,C). After LOX loading, the zeta potential of MnO_2_ nanoparticles decreased from ≈20 mV to ≈0 mV, forming stable noncovalent complexes (Figure 1D). The BCA assay determined that the LOX loading capacity of MnO_2_ nanoparticles was 54.18 ± 6.97%. EDS maping was used to verify the elemental distribution and showed that manganese (Mn) and oxygen (O) were evenly distributed in LM and MnO_2_ nanoparticles, while the percentage of nitrogen (N) was higher in LM group, indicating the loading of protein (Figure 1E). We performed X‐ray photoelectron spectroscopy (XPS) to further confirm the formation of the MnO_2_ and study the chemical valence of manganese element in LOX‐MnO_2_. As shown in Figure S3 (Supporting Information), two characteristic peaks at 653.5 and 642.5 eV could be assigned to the spin–orbit peaks of Mn (IV) 2p1/2 and Mn (IV) 2p3/2 of MnO_2_. SDS‒PAGE, followed by gel staining, showed LOX bands at ≈40 kDa and similar bands in LM, indicating successful loading of LOX on MnO_2_ nanoparticles (Figure 1F). UV/Vis spectra showed similar spectra between MnO_2_ and LM, suggesting that loading LOX barely changed the absorption property of MnO_2_ nanoparticles (Figure 1G).

### 2.2 Characteristics of LOX‐MnO_2_ Nanozyme‐Loaded dECM Microspheres

**Figure 2:**
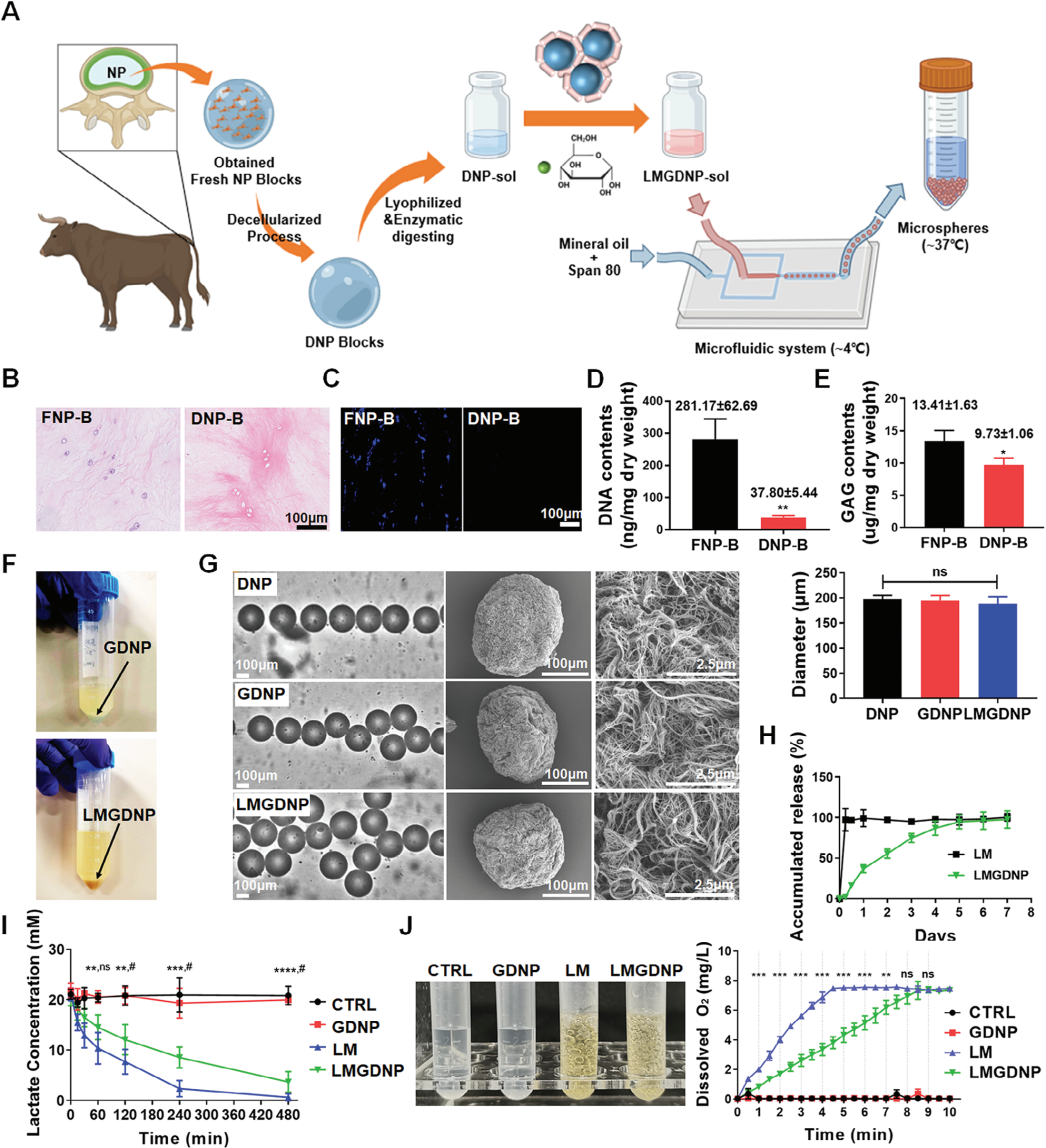
Preparation and characteristics of functional microspheres. A) Schematic overview of the construction of LMGDNPs. Micrographic images to evaluate the efficiency of decellularization based on B) HE staining and C) DAPI staining. Scale bar: 100 µm. D) Quantitative analysis of DNA content (ng/mg dry weight) in FNP‐B and DNP‐B. E) GAG analysis of FNP‐B and DNP‐B evaluating the content of proteoglycans (ng/mg dry weight). F) Collected microspheres dispersed in mineral oil. Data are presented as the mean ± SD, *n* = 3, *p < 0.05, **p < 0.01 between groups. G) Size distribution and microstructure of microspheres. Scale bars: 100 µm and 2.5 µm. Data are presented as the mean ± SD, *n* = 3, ns, no significance. H) Cumulative release profile of LOX in LMGDNPs based on the BCA assay. I) Lactate consumption efficiency of LOX‐MnO_2_ nanozyme and microspheres. Data are presented as the mean ± SD, *n* = 3, **p < 0.01, ***p < 0.001, ****p < 0.0001 between LM and CTRL groups; ns, no significance, ^#^ *p* < 0.05 between LM and LMGDNP groups. J) Dissolved oxygen profiles of H_2_O_2_ solution mixed with LOX‐MnO_2_ nanozyme and microspheres. Data are presented as the mean ± SD, *n* = 3, ns, no significance, **p < 0.01, ***p < 0.001 between LM and LMGDNP groups. NP, nucleus pulposus. CTRL, control. LM, LOX‐MnO_2_ nanozyme. DNP, decellularized nucleus pulposus matrix hydrogel‐based microspheres. GDNP, glucose‐rich nucleus pulposus matrix hydrogel‐based microspheres. LMGDNP, LOX‐MnO_2_ nanozyme‐loaded glucose‐rich nucleus pulposus matrix hydrogel‐based microspheres. FNP‐B, fresh nucleus pulposus tissue blocks. DNP‐B, decellularized nucleus pulposus tissue blocks.

NP tissues collected from bovine tails were decellularized to remove the cellular component and went through pepsin‐mediated enzymatic digesting to form the pregel solution, DNP‐sol. LMGDNP‐sol was generated by immersing genipin (0.02% w/v), LM (the mass ratio of LM nanoparticles: DNP = 1: 100) and glucose (5 mm) into DNP‐sol and was pumped into the microfluidic chip to form LMGDNP‐sol droplets by the shear force when encountering the oil flow in the intersectional region. LMGDNP‐sol droplets were further collected through a silicone tube immersed in a 37°C water bath, which allowed sol‐gel transition (**Figure** **2**A). HE and DAPI staining indicated that the cell nuclei were removed while the ECM remained after the decellularization process (Figure 2B,C). After decellularization, the DNA content decreased from 281.17 ± 62.69 ng mg^−1^ for FNP‐B to 37.80 ± 5.44 ng mg^−1^ for DNP‐B (Figure 2D), while glycosaminoglycan (GAG), comprising the major components of the ECM, decreased from 13.41 ± 1.63 µg mg^−1^ for FNP‐B to 9.73 ± 1.06 µg mg^−1^ for DNP‐B (Figure 2E), which suggested a significant removal of cellular components while retaining most of the ECM. The obtained microspheres immersed in the oil phase precipitated at the bottom. GDNPs appeared light blue in the presence of genipin, while LMGDNPs appeared brown due to MnO_2_ (Figure 2F). The diameters of the droplets were consistent (DNP: 197.63 ± 7.34 µm; GDNP: 195.49 ± 9.10 µm; LMGDNP: 187.94 ± 14.36 µm) under real‐time observation, and the nanofibrous structures of the collected microspheres were identical to those of the self‐assembled nanostructure of the DNP hydrogel (Figure 2G), providing an ECM‐mimicking microenvironment to support cell attachment, proliferation, and directed differentiation.^[^ ^24^, ^25^, ^26^ ^]^ To visualize the morphology changes of the LMGDNP during spontaneous degradation, microspheres were first immersed in PBS solution, before being dehydrated and dried at predetermined time points for SEM characterization. It was noted that the microgels maintained a spherical shape and a nanofibrous ultrastructure at least for the first 2 weeks postfabrication, indicating that the degradation was considerably slow (Figure S4, Supporting Information). Moreover, the loading capacity of MnO_2_ onto LMGDNPs was 9.31 mg g^−1^. Since the the loading efficiency of LOX onto MnO_2_ nanoparticles was 54.18 ± 6.97%, the resulted content of LM in LMGDNPs was calculated to be ≈9.43 mg g^−1^. Therefore, the loading efficiency of LM onto LMGDNPs were 95.2%. In addition, LMGDNPs showed a delayed release profile of LOX, as detected with the BCA assay (Figure 2H). The lactate concentration determined at time points showed a gradual decrease in both the LMGDNP and LM groups, but LMGDNPs seemed to be less effective than LM, which may be attributed to the delayed release of LOX into the enzymatic reactive system (Figure 2I). H_2_O_2_, generated from enzymatically consuming lactate, is a harmful byproduct that causes oxidative stress.^[^ ^27^ ^]^ The accumulating oxygen production in the LMGDNP and LM groups suggested a catalase‐like ability of LMGDNPs and LM in eliminating H_2_O_2_ and generating oxygen to improve the hypoxic microenvironment in IVD (Figure 2J). The delayed oxygen production in the LMGDNP group indicates a gradual release of LM, resulting in the relatively low concentration of MnO_2_ nanoparticles (Figure 2J). Besides, both GDNP and LMGDNP showed similar release pattern of glucose (Figure S5, Supporting Information).

### 2.3 Biocompatibility and Pro‐Differentiation Capacity of LMGDNPs

**Figure 3:**
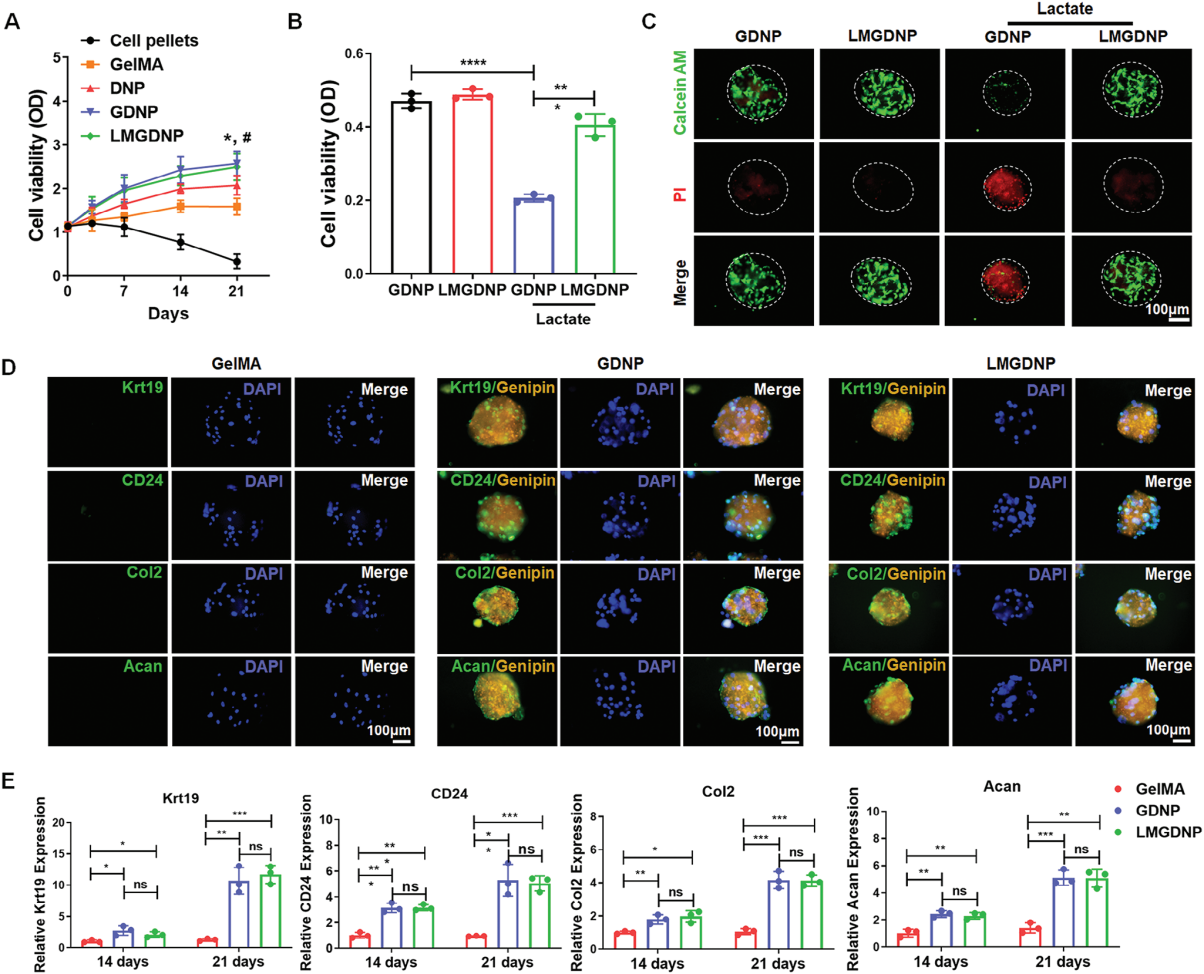
Pro‐differentiation capacity and bioactivity of LMGDNPs against lactate. A) CCK8 assay detecting the viability of BMSCs cultured in pellets or on microspheres. Data are presented as the mean ± SD, *n* = 3, ns, no significance, *p < 0.05 between DNP and GelMA group; #*p* < 0.05 between LMGDNP and DNP group. B) Evaluation of the viability of BMSCs cultured on microspheres with or without lactate for 24 h. C) Fluorescence images of the live/dead assays of BMSCs cultured on microspheres with or without lactate for 24 h. Scale bar: 100 µm. D) Representative immunocytochemistry images showing the expression of NPCs markers (Krt19, CD24, Col2, and Acan) in BMSCs cultured on microspheres for 21 days. Genipin was visualized using orange fluorescence. Scale bar: 100 µm. E) Relative mRNA expression of Krt19, CD24, Col2, and Acan in BMSCs cultured on microspheres for 14 and 21 days. Data are presented as the mean ± SD, *n* = 3, ns, no significance, *p < 0.05, **p < 0.01, ***p < 0.001 between groups. DNP, decellularized nucleus pulposus matrix hydrogel‐based microspheres. GDNP, glucose‐rich nucleus pulposus matrix hydrogel‐based microspheres. LMGDNP, LOX‐MnO_2_ nanozyme‐loaded glucose‐rich nucleus pulposus matrix hydrogel‐based microspheres.

BMSCs cultured on GelMA, DNP, GDNPs and LMGDNPs showed no obvious cell death on the 14th day, while PI‐positive staining was significantly increased in the cell pellets, especially for the core, which may be attributed to better nutrient and oxygen supply for cells cultured on the surface of microspheres (Figure S6, Supporting Information). The CCK8 assay indicated that the cell viability increased more obviously in DNP than GelMA and cell pellets (**Figure** **3**A), indicating that the dECM microspheres are more suitable for cell survival than GelMA. The GDNP and LMGDNP groups showed better cell viability than DNP group (Figure 3A), supported by the evidence that cell viability was improved with glucose supplementation (Figure S7, Supporting Information). The lactate concentrations in the inner annulus of LBP patients largely vary from 2 to 6 mm.^[^ ^15^ ^]^ To evaluate the capacity of microspheres to consume lactate, lactate (6 mm) was added to the microsphere‐culturing system. As a result, lactate impaired cell viability and caused more cell death in the GDNP group but exhibited no obvious impact on the LMGDNP group (Figure 3B,C). To evaluate the pro‐differentiation capacity of dECM microspheres, BMSCs were cultured on GelMA, GDNPs and LMGDNPs for 14 days and 21 days. On the 21st day, immunofluorescence staining of NPCs markers (Krt19, CD24, Col2, and Acan) showed significant positive staining in the GDNP and LMGDNP groups but not in the GelMA group (Figure 3D). Supportively, the mRNA expression of these markers was also significantly increased in the GDNP and LMGDNP groups (Figure 3E).

### 2.4 LMGDNPs Improve Cells Survival and Matrix Metabolism by Consuming Lactate

**Figure 4:**
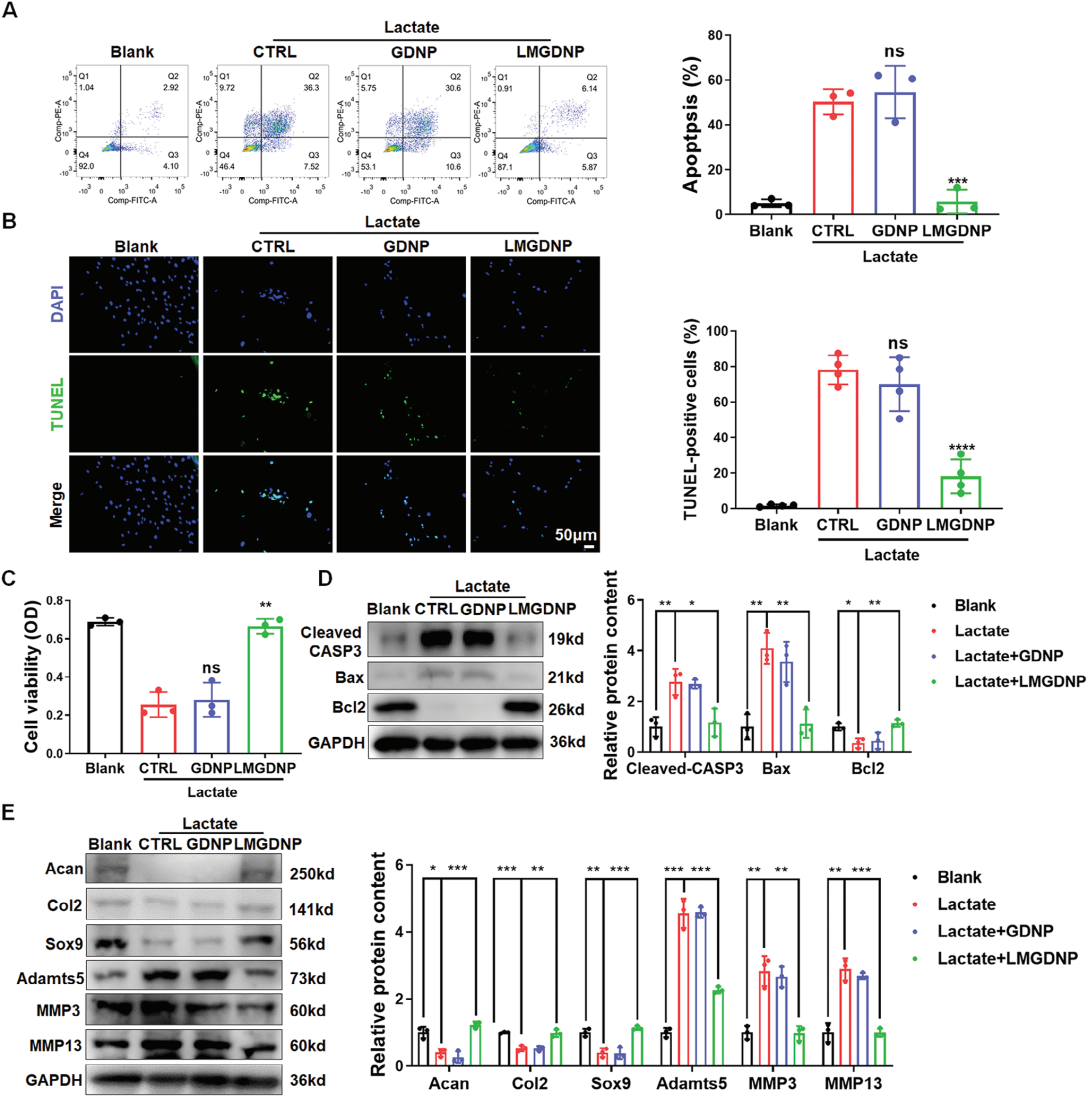
Anti‐lactate effects of LMGDNPs on cell survival and matrix regeneration. A) Flow cytometry results of Annexin V/PI staining of NPCs cocultured with microspheres with or without lactate treatment for 24 h. The apoptosis rate is the sum of the proportion of Annexin V^+^ PI^−^ and Annexin V^+^ PI^+^ cells. B) TUNEL staining of NPCs exposed to microspheres with or without lactate treatment for 24 h. Scale bar: 50 µm. The positive rate of TUNEL staining in green fluorescence was calculated and is shown in the subjacent chart. The results are shown as the mean ± SD, *n* = 4. C) The viability of NPCs cocultured with microspheres with or without lactate treatment. The results are shown as the mean ± SD, *n* = 3, ns, compared to lactate + CTRL; **p < 0.01, ***p < 0.001, ****p < 0.0001, compared to lactate + GDNP. D) Blots and densitometric analysis of apoptosis‐related proteins in NPCs treated with microspheres and lactate. E) Western blot analysis of matrix‐degrading proteases (Adamts5, MMP3, and MMP13) and matrix anabolic factors (Acan, Col 2, and Sox9). Data are presented as the mean ± SD, *n* = 3, ns, no significance, *p < 0.05, **p < 0.01, ***p < 0.001 between groups. CTRL, control. LM, LOX‐MnO_2_ nanozyme. GDNP, glucose‐rich nucleus pulposus matrix hydrogel‐based microspheres. LMGDNP, LOX‐MnO_2_ nanozyme‐loaded glucose‐rich nucleus pulposus matrix hydrogel‐based microspheres.

Lactate has been reported to cause cell death and lead to matrix degradation.^[^ ^16^, ^28^ ^]^ We evaluated the capacity of LMGDNPs to eliminate the harsh effects of lactate by a culture system in which NPCs were directly exposed to lactate and microspheres. With flow cytometry measuring annexin V‐PI‐stained cells, we found that the apoptosis rate was significantly increased in the lactate + CTRL group, as evidenced by an elevated proportion of Annexin ^+^ PI ^−^ and Annexin ^+^ PI ^+^ cells, while LMGDNPs downregulated the apoptotic rate, indicating the protective effects of LMGDNPs against the lactate‐enriched microenvironment (**Figure** **4**A). The TUNEL apoptosis assay also showed increased positive staining in the lactate group, while the number of TUNEL‐positive cells decreased in the LMGDNP group but not in the GDNP group, suggesting that the lactate‐consuming effects of LM prevented cell death (Figure 4B). Supportively, cell viability was also significantly improved by LMGDNPs (Figure 4C). In the presence of lactate, the anti‐apoptotic protein BCL2 decreased, while an increase in the pro‐apoptotic proteins BAX (BCL2 associated X, apoptosis regulator) and cleaved CASP3 was observed. LMGDNPs promoted the expression of BCL2 and downregulated the expression of BAX and cleaved CASP3 (Figure 4D). Moreover, LMGDNPs also blocked the effects of lactate on promoting the expression of major matrix‐degrading proteases (Adamts5, MMP3, and MMP13) and downregulating matrix anabolic factors (Acan, Col II and Sox9), indicating that lactate consumption maintained ECM metabolic balance and promoted matrix regeneration (Figure 4E).

### 2.5 The Regenerative Effect of LMGDNPs Relies on Autophagy Activation

**Figure 5:**
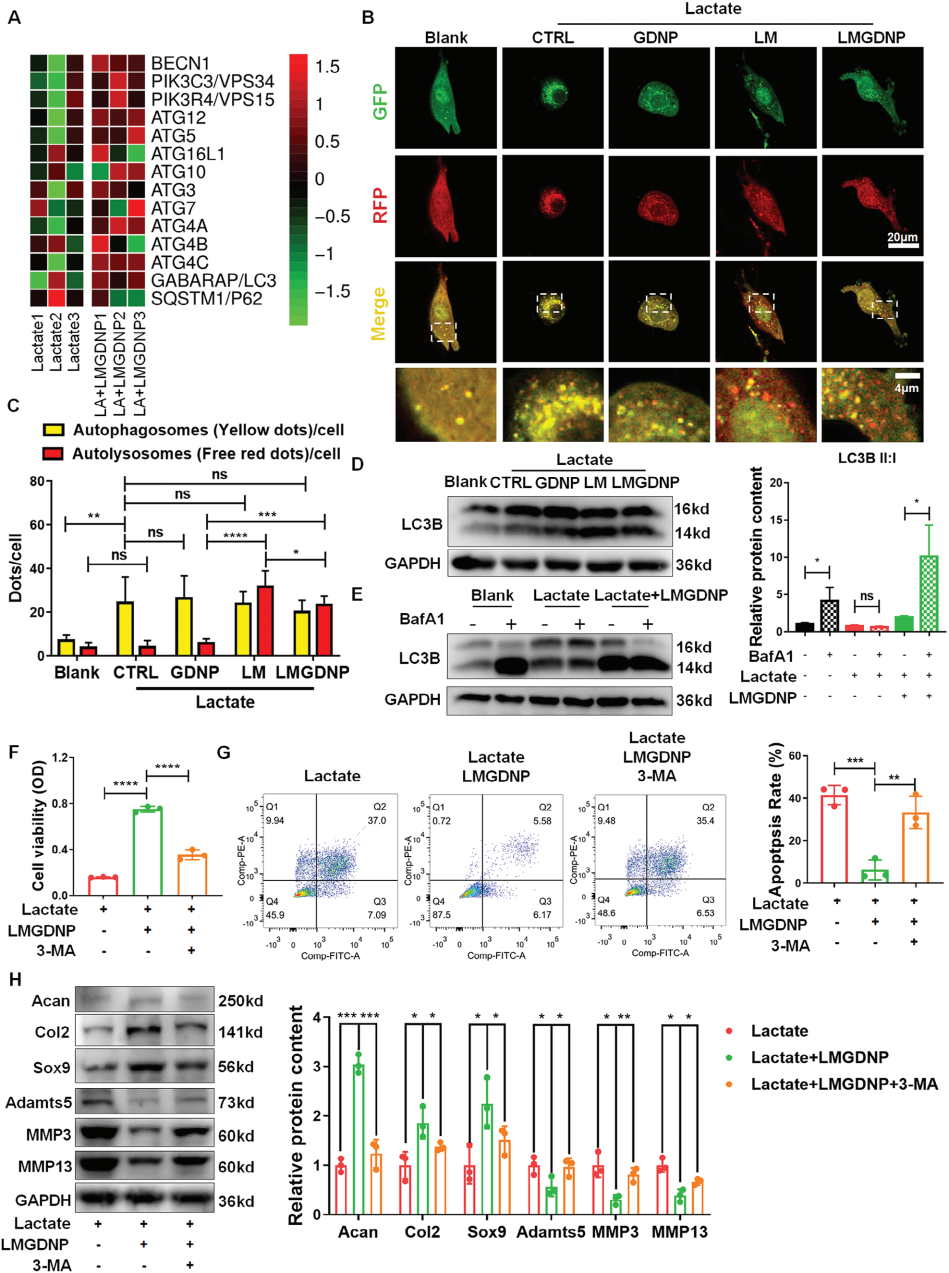
Autophagy mediated the anti‐apoptosis and matrix regeneration effects of LMGDNPs. A) Heatmaps for normalized gene expression of autophagy‐associated genes in NPCs cultured with or without LMGDNPs. B) Representative fluorescence images showing the autophagosomes (yellow dots, GFP+ RFP+) and autolysosomes (free red dots, GFP− RFP+) in NPCs exposed to lactate and GDNPs, LM, or LMGDNPs for 24 h. Scale bars: 20 µm and 4 µm. C) Quantification of autophagosomes and autolysosomes per cell is shown as the mean ± SD. Cells were from 3 independent experiments. ns, no significance; *p < 0.05, **p < 0.01, ***p < 0.001, ****p < 0.0001 between groups. D) Western blot analysis of LC3B in NPCs. E) Western blot analysis of LC3B‐II:I in NPCs treated with lactate and LMGDNPs for 24 h. BafA1 was added in the last 4 h. Densitometric analysis of LC3B‐II:I is shown in the statistical chart. F) The viability of cells exposed to lactate, LMGDNPs and 3‐MA. G) Cell flow cytometry analysis of the apoptotic rate of NPCs. H) Western blot analysis of matrix‐degrading proteases and matrix anabolic factors. Data are presented as the mean ± SD, *n* = 3, *p < 0.05, **p < 0.01, ***p < 0.001, ****p < 0.0001 between groups. LA, lactate. CTRL, control. LM, LOX‐MnO_2_ nanozyme. GDNP, glucose‐rich nucleus pulposus matrix hydrogel‐based microspheres. LMGDNP, LOX‐MnO_2_ nanozyme‐loaded glucose‐rich nucleus pulposus matrix hydrogel‐based microspheres. 3‐MA, 3‐Methyladenine.

To further investigate the mechanism, we analyzed the transcriptome of NPCs cultured with or without LMGDNPs in the presence of lactate (Figure S8, Supporting Information). We found that LMGDNPs upregulated the expression levels of several autophagy‐associated genes (BECN1, ATG12, 5, 10, 3, 7, 4, and GABARAP/LC3) (**Figure** **5**A). Autophagy plays a critical role in IVD cell survival, differentiation, and many other pathophysiological processes.^[^ ^29^, ^30^, ^31^ ^]^ Thus, we evaluated the activation of autophagy in NPCs. mRFP‐GFP‐LC3 adenovirus (AdV‐mRFP‐GFP‐LC3) was used to transfect NPCs to visualize the autophagosomes and autolysosomes. When LC3‐label fluorescence is merged from both RFP and GFP, yellow dots, indicating the formation of autophagosomes, can be identified. As the autophagic flux continues and autolysosomes increase, the proportion of red dots increases since the acidic lysosomes can quench GFP. We found a significant increase in the number of autophagosomes in the lactate + CTRL and lactate + GDNP groups but no obvious alteration of autolysosomes, suggesting a blockage of autophagic flux (Figure 5B,C). Surprisingly, the LM and LMGDNP groups did not block the effects of lactate on promoting the formation of autophagosomes but further increased the number of autolysosomes, indicating that the blockage of autophagic flux by lactate was relieved and autophagy was activated in the LM and LMGDNP groups (Figure 5B,C). Therefore, LMGDNPs may inhibit the blocking effects of lactate on autophagic flux by eliminating lactate, and further promoting autophagy activation. Supportively, although the lactate + CTRL and lactate + GDNP groups showed higher LC3B expression than the Blank group, the LC3B‐II:I ratio decreased in these groups, indicating the blockage of autophagic flux. The LC3B‐II:I ratio in the LM and LMGDNP groups increased by 1.8‐fold compared to that in the lactate + GDNP group, which verified that LM contributed to the activation of autophagy (Figure 5D; Figure S9, Supporting Information). Bafilomycin A 1 (BafA1), which prevents the fusion of autophagosom es and lysosomes, was used to analyze the influx of autophagy. The LC3B‐II:I ratio showed no obvious change with the administration of BafA1 in the lactate group, indicating the blockade of autophagic flux. The lactate + LMGDNP group showed a higher LC3B‐II:I ratio than the lactate group, and BafA1 caused a further increase in the LC3B‐II:I ratio in the lactate + LMGDNP group, suggesting that LMGDNP had an autophagy‐stimulating effect (Figure 5E).

3‐Methyladenine (3‐MA) (5 mm) was applied to inhibit the activation of autophagy. Consequently, the improved cell viability in the lactate + LMGDNP group was impaired in the presence of 3‐MA (Figure 5F). The proportion of apoptotic NPCs increased when 3‐MA was added to the lactate + LMGDNP culture system (Figure 5G). Supportively, the anti‐apoptotic protein BCL2 was downregulated, while an increase in BAX and cleaved CASP3 was observed in the 3‐MA + lactate + LMGDNP group compared with the lactate + LMGDNP group (Figure S10, Supporting Information). These results suggest that the anti‐apoptotic effects of LMGDNPs partially relied on the activation of autophagy. Furthermore, the expression of Acan, Col2, and Sox9 was significantly downregulated, while Adamts5, MMP3, and MMP13 levels increased when autophagy was inhibited, suggesting that the matrix regenerative capacity of LMGDNPs also partially relied on autophagy activation (Figure 5H).

### 2.6 TGFB2‐OT1 Mediates LMGDNPs‐Induced Autophagy Activation

**Figure 6:**
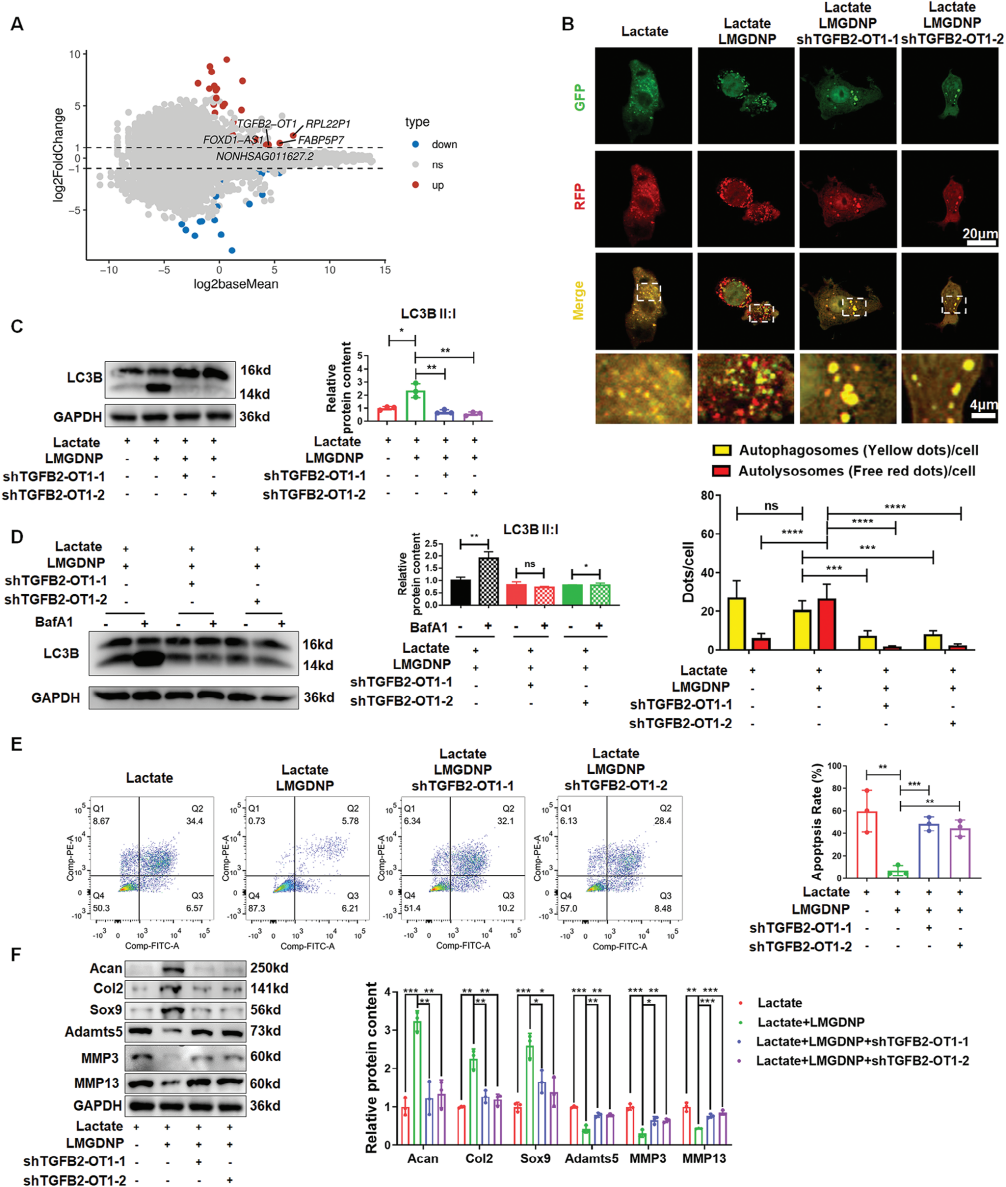
TGFB2‐OT1 mediates LMGDNPs‐induced autophagy in NPCs. A) Maplot of differentially expressed genes between the lactate and lactate + LMGDNP groups. MA plots (M = log_2_ fold change and A = log_2_ baseMean average). Red and blue dots in the MA plots indicate differentially upregulated and downregulated genes (*p* < 0.05) in NPCs cultured with LMGDNPs or not. The top 5 upregulated genes with the highest expression abundances are labeled. B) Representative fluorescence images showing the autophagosomes (yellow dots, GFP+ RFP+) and autolysosomes (free red dots, GFP− RFP+) in NPCs transfected with shTGFB2‐OT1 and exposed to lactate and LMGDNPs for 24 h. Scale bars: 20 µm and 4 µm. Quantification of the number of autophagosomes and autolysosomes per cell is shown as the mean ± SD. Cells were from 3 independent experiments. ns, no significance; ***p < 0.001, ****p < 0.0001 between groups. C) Western blot analysis of LC3B in NPCs transfected with shTGFB2‐OT1, which were treated with lactate and LMGDNPs for 24 h. D) LC3B II:I expression in NPCs transfected with shTGFB2‐OT1, which were treated with lactate and LMGDNPs for 24 h and cultured with or without BafA1 for 4 h. E) Cell flow cytometry analysis of the apoptotic rate of NPCs. The results are shown as the mean ± SD, *n* = 3, ***p < 0.001, ****p < 0.0001. F) Western blot analysis of matrix‐degrading proteases and matrix anabolic factors. Data are presented as the mean ± SD, *n* = 3, ns, no significance, *p < 0.05, **p < 0.01, ***p < 0.001 between groups. GDNP, glucose‐rich nucleus pulposus matrix hydrogel‐based microspheres. LMGDNP, LOX‐MnO_2_ nanozyme‐loaded glucose‐rich nucleus pulposus matrix hydrogel‐based microspheres.

After differential gene expression analysis of NPCs cultured with or without LMGDNPs, we found 103 differentially expressed genes with an expression change of >2 (fold change) and a p value of <0.05 (**Figure** **6**A). Among the top five with the highest average expression levels among the 53 upregulated genes, we identified an autophagy‐associated long noncoding RNA (lncRNA), TGFB2‐OT1, that was highly expressed in the LMGDNP group and has previously been reported to mediate the expression of key modulators (ATG13, ATG3 and ATG7) of autophagosomes by targeting microRNA 4459 (miRNA 4459).^[^ ^32^ ^]^ In addition, TGFB2‐OT1 also blocked miRNA 3960 and miRNA 4488 and affected their target genes (ceramide synthase 1 (CERS1) and N‐acetyltransferase 8‐like (GCN5‐related, putative) (NAT8L)), respectively) that regulate mitochondria‐related autophagy.^[^ ^33^, ^34^ ^]^ Therefore, TGFB2‐OT1 may play an important role in LM‐mediated autophagy activation. Supportively, TGFB2‐OT1 expression was significantly increased in both the lactate + LM and lactate + LMGDNP groups compared with the lactate and GDNP group (Figure S11, Supporting Information). Lentivirus transfection of shTGFB2‐OT1 was applied to knock down the expression of TGFB2‐OT1 (Figure S12, Supporting Information). As a result, the numbers of autophagosomes and autolysosomes were both downregulated after TGFB2‐OT1 knockdown (Figure 6B). The expression of LC3B‐II:I was also significantly reduced when NPCs were transfected with shTGFB2‐OT1 (Figure 6C). Supportively, BafA1 added to NPCs transfected with shTGFB2‐OT1 caused no significant increase in the LC3B‐II:I ratio, indicating the blockage of autophagic flux after downregulating TGFB2‐OT1 (Figure 6D). These results suggest that LMGDNP‐induced autophagy activation was mediated by TGFB2‐OT1.

Moreover, cell viability was also impaired, and the apoptotic rate increased significantly in the lactate+LMGDNP+shTGFB2‐OT1 group compared with the lactate + LMGDNP group, suggesting that the protective effects of LMGDNP against lactate accumulation on NPCs survival were inhibited by TGFB2‐OT1 knockdown (Figure 6E; Figure S13A, Supporting Information). As expected, the proapoptotic proteins BAX and cleaved CASP3 were increased, while BCL2 was downregulated after knocking down TGFB2‐OT1 (Figure S13B, Supporting Information). Proteins that mediate ECM degradation (Adamts5, MMP3, and MMP13) were also increased, and matrix proteins (Acan, Col2, and Sox9) were decreased in NPCs transfected with TGFB2‐OT1 (Figure 6F). The TGFB2‐OT1 downstream pathway was also evaluated. We found that miRNA 4459, miRNA 3960, and miRNA 4488 were all downregulated in the lactate + LMGDNP group compared with the lactate group and were upregulated when TGFB2‐OT1 was knocked down (Figure S14A, Supporting Information). The protein expression of CERS1, NAT8L, ATG13, ATG3, and ATG7 was upregulated by LMGDNPs but decreased by shTGFB2‐OT1 (Figure S14B, Supporting Information). Therefore, LMGDNPs induced autophagy activation via TGFB‐OT1 and its downstream pathway.

### 2.7 Stem Cells‐Loaded LMGDNPs Promote In Vivo Disc Regeneration

**Figure 7:**
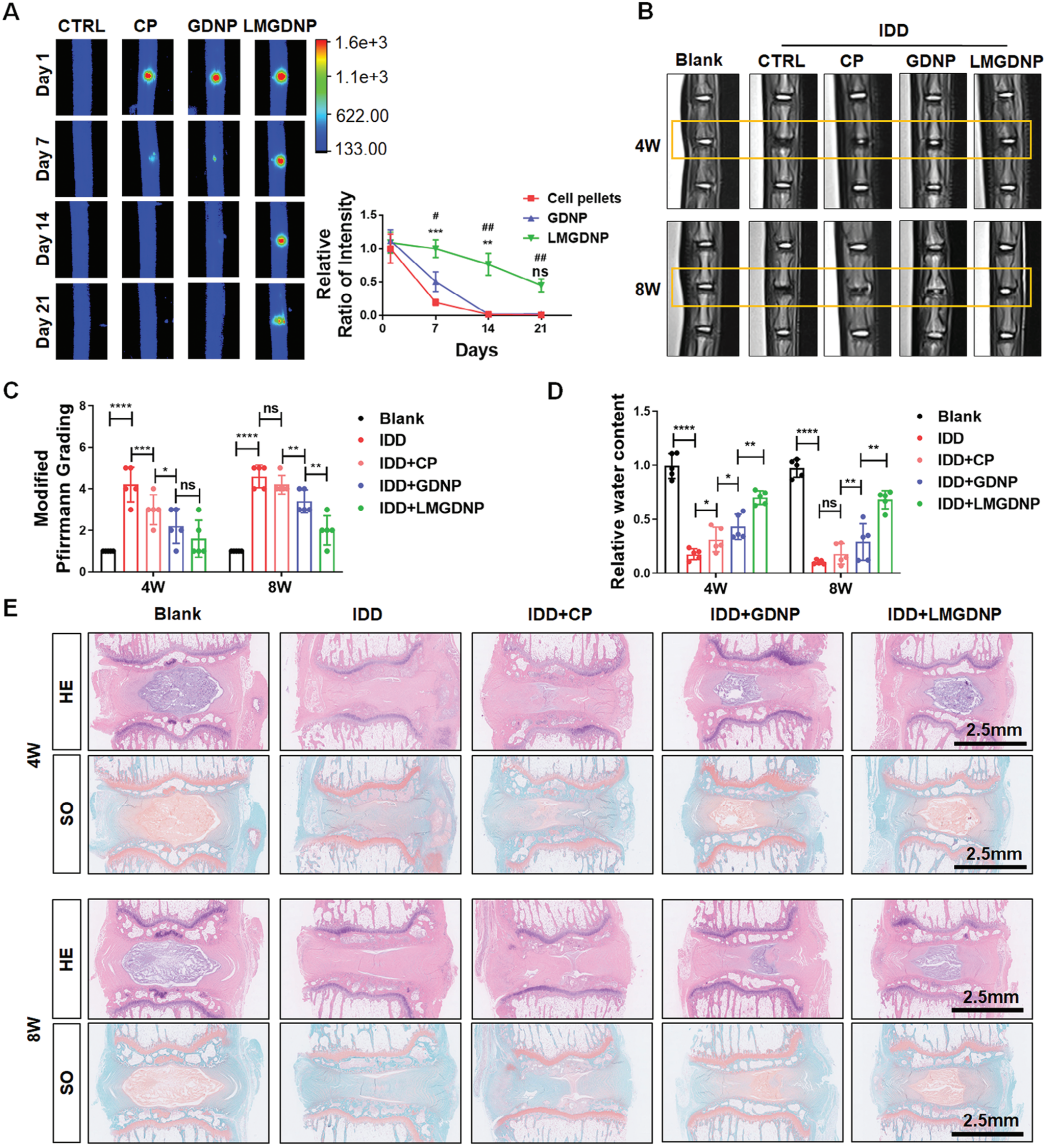
Regenerative effects of LMGDNPs on lactate‐induced IDD. A) Fluorescence images showing the fluorescence intensity after delivering DiR Iodide‐labeled BMSCs by GDNPs or LMGDNPs at Day 1, Day 7, Day 14, and Day 21. Data are presented as the mean ± SD, *n* = 3. Ns, no significance; **p < 0.01, ***p < 0.001, GDNPs compared to cell pellets (CP); #*p* < 0.05, ##*p* < 0.01, LMGDNPs compared to CP. B) Representative images of T2‐weighted MRI of rat tails. Boxes indicate the operated disc. C) Modified Pfirrmann MRI grades for each group. D) Quantitative evaluation of T2‐weighted signaling (water preservation in NP tissues). E). Histological images based on HE and SO staining. Scale bar: 2.5 mm. Data are presented as the mean ± SD, *n* = 5, ns, no significance, *p < 0.05, **p < 0.01, ***p < 0.001, ****p < 0.0001 between groups. CTRL, control. CP, cell pellets. IDD, intervertebral disc degeneration. LM, LOX‐MnO_2_ nanozyme. GDNP, glucose‐rich nucleus pulposus matrix hydrogel‐based microspheres. LMGDNP, LOX‐MnO_2_ nanozyme‐loaded glucose‐rich nucleus pulposus matrix hydrogel‐based microspheres.

**Figure 8:**
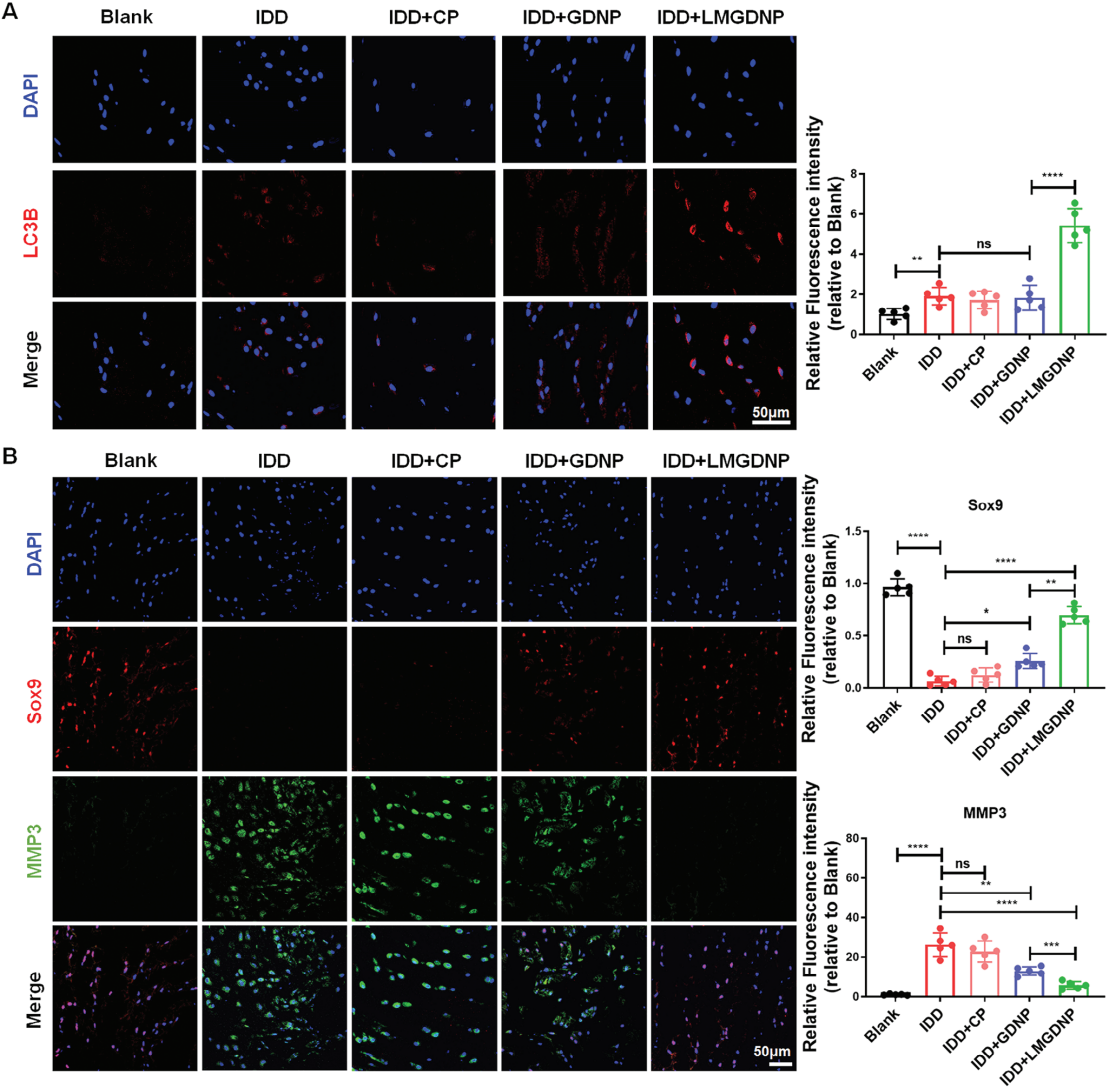
LMGDNPs promote autophagy and maintain matrix metabolism balance. A) Immunofluorescence detection of LC3B. B) Representative immunofluorescence images of Sox9 (red) and MMP3 (green). Scale bars, 50 µm. Data are presented as the mean ± SD, *n* = 5, ns, no significance, *p < 0.05, **p < 0.01, ***p < 0.001, ****p < 0.0001 between groups. CP, cell pellets. IDD, intervertebral disc degeneration. LM, LOX‐MnO_2_ nanozyme. GDNP, glucose‐rich nucleus pulposus matrix hydrogel‐based microspheres. LMGDNP, LOX‐MnO_2_ nanozyme‐loaded glucose‐rich nucleus pulposus matrix hydrogel‐based microspheres.

Lactate solution was injected into rat Co5/6 cells to establish the IDD model. One week later, normal saline, BMSC pellets, BMSCs‐loaded GDNPs and LMGDNPs were injected into the operated discs via a 29‐gauge needle. The supplementary lactate was injected 4 weeks after the first injection, and histological and radiological evaluation was performed at the 4^th^ week and 8^th^ week after the first lactate injection. The survival of DiR Iodide‐labeled BMSCs implanted into the discs was visualized by the IVIS Lumina Imaging Systems. We identified the slowest decay of DiR iodide signaling in the LMGDNP group compared with the other groups, and approximately 45% of BMSCs could still be detected on the 21st day (**Figure** **7**A). However, signaling in the CP and GDNP groups dissipated within 14 days (Figure 7A). Besides, histological staining showed that the percentage of DiR+/DAPI+ cells in disc tissues was the highest, and the percentage of TUNEL+ cells was the lowest in LMGDNP group (Figure S15, Supporting Information). MRI T2 phase images showed that the NP intensity was significantly improved in the GDNP and LMGDNP groups at the 4th week, while LMGDNPs mostly retained NP intensity at the 8th week (Figure 7B). The modified Pfirrmann grading system based on MRI images evaluates the severity of IDD. The grading showed that LMGDNPs mostly alleviated IDD at the 8th week (Figure 7C). Quantitation of the water content showed a significant loss of NP hydration in the IDD group, and implantation of BMSC pellets and BMSCs‐loaded GDNPs mildly improved NP hydration at the 4th week. BMSC pellets showed no significant improvement at the 8th week, while BMSCs‐loaded LMGDNPs significantly preserved NP hydration at the 4th week, and the most significant preservation was identified in the LMGDNP group at the 8th week (Figure 7D). Histological grading based on HE and SO staining showed that the NP tissues in the IDD group were shrunken and irregularly shaped, whereas BMSCs‐loaded LMGDNPs significantly alleviated morphological aggravation. Histological grade showed that BMSCs‐loaded LMGDNPs mostly reduced the severity of disc degeneration compared with other groups, especially at the 8th week (Figure 7E; Figure S16, Supporting Information). Moreover, LMGDNPs significantly promoted the expression of LC3B in NP tissues, suggesting activation of autophagy in vivo (**Figure** **8**A). MMP3 (a matrix‐degrading protease), Sox9 (an essential transcription factor that maintains extracellular matrix (ECM) homeostasis), and matrix proteins (Acan and Col2) were evaluated to determine matrix metabolism. Immunofluorescence showed that BMSCs‐loaded LMGDNPs significantly promoted Sox9 expression and inhibited MMP3 expression, indicating improved matrix anabolism (Figure 8B; Figure S17, Supporting Information). These results suggest that LMGDNPs promoted BMSCs survival after implantation and are an ideal cell delivery system for stem therapy for IVD regeneration.

## 3 Discussion

Although stem cell therapy and endogenous repair have shown great potential in IVD regeneration, many concerns have been stressed regarding the damage imposed on cells from the harsh environment in IDD.^[^ ^13^ ^]^ After exogenous stem cells are administered into the IVD, the energy requirement of the cells increases, while nutrients are relatively deficient for both implanted cells and endogeneous cells.^[^ ^35^ ^]^ Therefore, the imbalance of energy supply and demand is further exacerbated, leading to increased cell death and impaired cellular activity.^[^ ^36^ ^]^ Nevertheless, cell delivery systems designed for IVD regeneration barely target the unfavorable microenvironment of the IVD to improve the survival of cells. In this study, we specifically designed a glucose‐enriched delivery system that increased the local O_2_ concentration while exhausting lactic acid and improved cellular energy metabolism, thereby promoting the survival and function of both implanted MSCs and residual disc cells. In addition, the microspheres prepared from dECM hydrogels not only promoted cell differentiation toward NPCs but also served as carriers for LOX‐MnO_2_ nanozymes to maintain their local enrichment and enzymatic activity.

dECM hydrogels exhibit good biological activity and can promote cell survival, proliferation, and differentiation toward the corresponding tissue cells.^[^ ^37^ ^]^ Besides, the decellularization process for fabricating dECM removed the majority of DNA in the tissue, which reduced the risk of inflammative response. After decellularization, the remained DNA was 37.80 ± 5.44 ng mg^−1^ of dry weight in our study. Our previous research showed that DNA of 250 µg mL^−1^ could induce significant inflammatory reactions in macrophages.^[^ ^38^ ^]^ A study that evaluated the consequences of ineffective decellularization on the host response showed that DNA of 62 ± 16.4 ng mg^−1^ ECM exhibited minor pro‐inflammatory induction of macrophage, compared with that of 423 ± 29.7 ng mg^−1^.^[^ ^39^ ^]^ Using a rat partial thickness abdominal wall defect model, the authors found that a prolonged M1 response was observed surrounding the scaffold of higher DNA content at day 28. Conversely, the macrophages within the scaffolds with lower DNA content were much more evenly distributed, suggestive of a diminished pro‐inflammatory response to the scaffold material.^[^ ^39^ ^]^

To ensure their injectability, the hydrogels must be injected into the IVD in its solution phase before gelation is completed. To our knowledge, the gelation of dECM hydrogels mostly relies on neutral pH, isotonic and appropriate temperature.^[^ ^40^, ^41^, ^42^, ^43^ ^]^ However, the complex microenvironment in vivo, especially the low pH environment in IVD tissues, may impair the gel‐forming properties of dECM hydrogels in vivo.^[^ ^44^ ^]^ Microspheres, on the other hand, allow gelation in a controlled system while retaining their injectability by controlling particle size, which is more conducive to clinical applications.^[^ ^45^ ^]^ However, the weak mechanical properties and significant inhomogeneity of dECM compared to other natural biomaterials (e.g., gelatin, alginate, chitosan, etc.) brought great difficulties in the fabrication of dECM‐based hydrogel microspheres.^[^ ^23^ ^]^ We established a microfluidic system by which dECM microspheres were prepared with the aid of chemical crosslinking. The microspheres showed uniform size, appropriate porosity, and fiber structure. In addition, MSCs adhered well to the surface of the microspheres, which maintained cell activity and proliferation and promoted the directional differentiation of MSCs to NPCs.

Glucose is one of the critical nutrients for the survival of stem cells and IVD cells.^[^ ^46^ ^]^ The concentration of glucose in normal human discs was measured as 1.25 mm and further reduced to approximately 0.5 mm due to calcified endplates and diminished vertebral blood supply in IDD.^[^ ^47^, ^48^, ^49^ ^]^ As the concentration of glucose increased from 0.5 to 5 mm, cell viability and proliferation were significantly improved.^[^ ^47^ ^]^ Additional supplementation with glucose may be a feasible approach to increase the nutrient supply for implanted MSCs and IVD cells. We found that glucose supplementation provided more nutrients for energy production, which led to better cell viability and increased matrix synthesis.^[^ ^47^ ^]^

Lactate, the end‐product of glycolysis, has been recognized as a biomarker of IDD since 1968 and has been recently suggested as a biomarker of discogenic pain.^[^ ^50^, ^51^ ^]^ We found that the LOX‐MnO_2_ nanozyme achieved efficient clearance of lactate, resulting in a significant inhibitory effect against lactate‐induced cell apoptosis. In addition, LM significantly promoted cellular autophagy. Autophagy is a complex catabolic pathway that maintains cellular homeostasis by mediating the degradation of a variety of intracellular materials ranging from proteins to organelles^[^ ^52^ ^]^ through autophagosomes, including macroautophagy, microautophagy, and chaperone‐mediated autophagy.^[^ ^53^, ^54^ ^]^ Macroautophagy is the most common type of autophagy.^[^ ^54^ ^]^ When macroautophagy is initiated, the endoplasmic reticulum triggered by autophagy activators starts to form an omegasome that matures and elongates to shape a double‐membrane vesicle named an autophagosome.^[^ ^55^ ^]^ Then, the autophagosome and lysosome are fused to form autolysosomes to decompose the substrate encapsulated in the autophagosome by lysosomal hydrolase.^[^ ^55^ ^]^ Autophagy plays a critical role in the pathophysiological process of IVD cells.^[^ ^30^ ^]^ The enhancement of autophagy significantly prevents ECM degradation by reducing the expression of catabolic factors such as MMPs.^[^ ^56^, ^57^ ^]^ We have previously reported that the activation of autophagy mediates the anti‐apoptotic effect of HIF1A against compression stress.^[^ ^29^ ^]^ In this study, we found that although the expression of autophagy protein (LC3B) was increased in the lactate group, autophagic flow was blocked. LOX‐MnO_2_ nanozyme alleviated the blockade of autophagic flow imposed by lactate and, surprisingly, further increased the levels of LC3B and LC3B II:I. This indicated that, in addition to consuming lactate, the LOX‐MnO_2_ nanozyme could further promote autophagy.

Increasing evidence has suggested the critical role of the lysosome‐autophagy system in the cell's adaptive reaction to nanomaterials.^[^ ^58^, ^59^ ^]^ Nanomaterial‐induced autophagy may be determined by means of nanosize, physiochemical properties, and composites.^[^ ^58^, ^59^ ^]^ Zhang et al. compared Au nanoparticles with different diameters, among which 45 nm Au nanoparticles mostly promoted the level of autophagy and induced osteogenesis.^[^ ^60^ ^]^ In addition, researchers have investigated the activation of autophagy upon cells exposure to quantum dots and revealed that size‐dependent uptake of quantum dots is mediated by autophagy.^[^ ^61^, ^62^ ^]^ Interestingly, carbon nanotubes functionalized with surface ligands induced autophagy, while autophagy induction was not observed when cells were treated only with the ligands, suggesting that autophagy is activated in response to uptake of the nanomaterials and that the mode of interaction between the nanomaterial and the lysosome‐autophagy system determines the specific nature of the autophagic response.^[^ ^63^ ^]^ We found that the particle size of LM was ≈64 nm, which may activate the lysosome‐autophagy system in the cellular response to nanoparticles.^[^ ^61^, ^62^ ^]^

**Figure 9:**
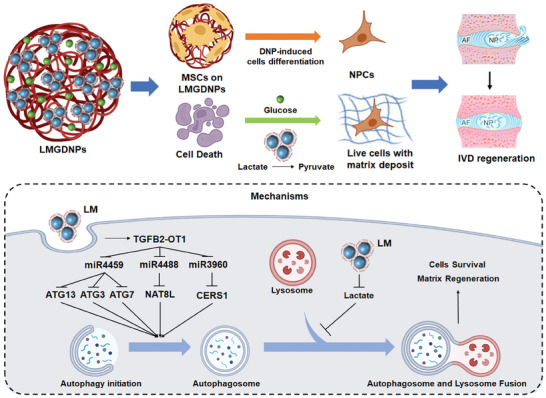
Schematic diagram of the effect and mechanism of LMGDNPs in alleviating IDD. LMGDNPs, as carriers of MSCs, induce directional differentiation of MSCs into NPCs; LMGDNPs reduce cell death and ECM breakdown by consuming lactate; LM released from LMGDNPs promotes the formation of autophagosomes through the TGFB2‐OT1/miRNAs/ATGs/NAT8L/CERS1 pathway, and consumes lactate to inhibit the blocking effect of lactate on autophagy flow, thereby activating autophagy, promoting cell survival and matrix regeneration.

To further identify the mechanism of LOX‐MnO_2_ nanozyme‐induced autophagy, we performed transcriptome sequencing, identified the key molecule that mediated LMGDNP‐induced autophagy, and demonstrated that LMGDNPs promoted NPCs autophagy by activating TGFB2‐OT1. TGFB2‐OT1, located in the 3′ untranslated region (3′UTR) of transforming growth factor β2 (TGFB2), is a long noncoding RNA (lncRNA) that sequesters miRNA 4459 and increases the level of the miRNA 4459 targets (ATG13 and LARP1).^[^ ^32^, ^64^ ^]^ LARP1 then promotes the expression of ATG3 and ATG7. ATG13, ATG3 and ATG7 are the key modulators of autophagosomes.^[^ ^32^, ^65^ ^]^ Therefore, TGFB2‐OT1 mediated the activation of autophagy by elevating ATG13, ATG3 and ATG7 expression by targeting miRNA 4459. In addition, TGFB2‐OT1 also acts as a ceRNA that blocks the effects of miRNA 3960 and miRNA 4488, which target ceramide synthase 1 (CERS1) and N‐acetyltransferase 8‐like (GCN5‐related, putative) (NAT8L), respectively.^[^ ^32^ ^]^ CERS1 and NAT8L regulate autophagy by affecting mitochondria.^[^ ^33^, ^34^ ^]^ Supportively, we observed the upregulation of ATG3, ATG7, ATG13, NAT8L, and CERS1 and the downregulation of miRNA 3960, miRNA 4488 and miRNA 4459 in the LM and LMGDNP groups. In addition, after knocking down TGFB2‐OT1, the formation of autophagosomes was significantly inhibited. Therefore, LM activated autophagy by alleviating the blockade of autophagic flux by consuming lactate and promoting the formation of autophagosomes by activating the TGFB2‐OT1/miRNAs/ATGs/NAT8L/CERS1 pathway (**Figure** **9**).

## 4 Conclusion

In this study, we fabricated glucose‐enriched microspheres consisting of LOX‐MnO_2_ nanozymes and dECM hydrogels as a stem cell delivery system for IDD therapy using microfluidic technology. These injectable microspheres showed uniform size, good biocompatibility, suitable microstructure, and excellent enzymatic activity against lactate. LMGDNPs significantly alleviated the pro‐apoptotic and matrix pro‐degradative effects of lactate. Moreover, LMGDNPs activated autophagy by promoting TGFB2‐OT1 to form autophagosomes and eliminating lactate to unblock autophagic flow. Furthermore, microspheres, composed of the ECM from native nucleus pulposus, promoted the tissue‐specific differentiation of stem cells for matrix synthesis and IVD regeneration. These glucose‐enriched dECM microspheres, functionalized by LOX‐MnO_2_ nanozymes, provide a novel strategy for stem cell delivery by supplying nutrients and eliminating the harsh effects of glycolytic products to achieve better cell survival and therapeutic effects against IDD.

## 5 Experimental Section

### Preparation and Characteristics of LOX‐MnO_2_ Nanozyme (LM)

MnO_2_ nanoparticles were prepared using an in situ crystallization method as reported in the literature with some modifications.^[^ ^66^ ^]^ Briefly, 64 mg of KMnO_4_ (Aladdin, China) was dissolved in 18 mL of DI water. Then, 2 mL of poly(allylamine hydrochloride) (PAH) (Aladdin, China) solution with a concentration of 36 mg mL^−1^ was dropped into the mixture. After reaction at 25 °C for 10 min, the obtained mixture containing MnO_2_ nanoparticles was dialyzed against DI water in a dialysis bag (molecular weight cutoff 3 kDa) for one week and then freeze‐dried to obtain lyophilized powder to calculate the yield. To prepare the LOX‐MnO_2_ nanoparticles (LM), LOX (50 U, Sigma‐Aldrich, USA) was added into 1 mL of the MnO_2_ nanoparticle dispersion (3.6 mg mL^−1^) under stirring (200 rpm) at 25°C for 30 min. Then, LMs were collected using centrifugation at 20 000 rpm for 30 min and washed with PBS three times.

The LOX loading capacity of MnO_2_ was determined with a bicinchoninic acid (BCA) assay and calculated as follows: loading capacity % = M_1_/M_0_ × 100% (M_0_: initial LOX content; M_1_: LOX content in LM).

The microstructures of the nanoparticles were determined using a Hitachi HT 7700 transmission electron microscope (TEM, HT7800, Hitachi, Japan) at 120 kV.

The major element distribution of MnO_2_ and LOX‐MnO_2_ nanozyme (LM) was elucidated using energy‐dispersive spectroscopy (EDS) mapping (SU8100, Hitachi, Japan).

The ultraviolet‒visible (UV‒Vis) spectra were determined using a UV‒vis spectrometer (UV‐2600, SHIMADZU, Japan).

The loading of LOX on MnO_2_ nanoparticles was assessed by means of a sodium dodecyl sulfate‒polyacrylamide gel electrophoresis assay (SDS‒PAGE) (Beyotime, China), as previously reported.^[^ ^20^ ^]^ Briefly, five mg LM was denatured using SDS‒PAGE loading buffer and heated for 10 min. Then, the supernatants were loaded onto 10% SDS‒PAGE gels for electrophoresis and illuminated using the Fast Silver Stain Kit (Beyotime, China). The particle size and zeta potential of the nanoparticles were measured using dynamic light scattering (DLS) (Zetasizer Advance Range, Malvern, UK).

### Preparation and Characteristics of Glucose‐Enriched Decellularized Nucleus Pulposus Microspheres Loaded with LOX‐MnO2 Nanozyme (LMGDNPs)

All procedures involving animals were approved by the Animal Experimentation Committee of Huazhong University of Science and Technology and followed the ARRIVE guidelines. Nucleus pulposus tissues were harvested from the caudal segments of bovines within 6 h of sacrifice from the abattoir and cut into small blocks, denoted fresh nucleus pulposus blocks (FNP‐B). Tissues were decellularized following previously described protocols.^[^ ^24^ ^]^ Briefly, NP tissues were sequentially decellularized with Triton X‐100 (2%), sodium dodecyl sulfate (SDS, 1%) and DNAase (Sigma, 200 U mL^−1^ in PBS). Then, processed tissues were lyophilized, forming decellularized nucleus pulposus blocks (DNP‐B). The DNP‐B blocks were homogenized using a Thomas Wiley Mini‐Mill (Thomas Scientific, Swedesboro, NJ, USA). After washing with sterile water, the powder was then digested in pepsin (0.1% w/v) containing HCl (0.01 m) at a concentration of 1% w/v for 48 h at 25°C. Then, the digestive solution was centrifuged to remove the undissolved particles. The pH of the digested solution was adjusted to 7.3–7.5 using 0.1 m NaOH, and the ionic strength was adjusted to an isotonic state to form the pregel solution (DNP‐sol). Glucose solution was added to DNP‐sol to obtain GDNP‐sol at a concentration of 5 mm. LM nanoparticles were added into GDNP‐sol to obtain LMGDNP‐sol by the mass ratio of LM nanoparticles: DNP = 1: 100. Then, the pregel solution was stored at 4°C until use.

The fabrication of microspheres followed our previously reported protocols using a microfluidic device.^[^ ^23^ ^]^ Briefly, after being designed using AutoCAD software (Autodesk, California, USA), the silicon master was fabricated using photolithography. Then, the Polydimethylsiloxane (PDMS, Dow Corning, USA) base was mixed with a curing agent and degassed for 15 min before being poured onto the silicon master. Next, after being degassed for another 2 h, the PDMS mold was cured at 70°C for 4 h and peeled off from the silicon master. Two 1.0 mm diameter wells for inflow channels and a 2.0 mm diameter well for the outflow channel were introduced on the PDMS chip. Meanwhile, a flat PDMS coversheet was prepared by a flat silicon wafer. Finally, after oxygen plasma treatment (30 W, PDC‐MG, MING HENG, China), the PDMS coversheet was carefully placed on the chip and placed at 70°C for 30 min to obtain the microfluidic chip. Then, the microspheres, denoted as DNPs, GDNPs, and LMGDNPs, were fabricated using a novel two‐stage temperature‐controlling microfluidic system (TSTC‐MS) (Figure S1, Supporting Information). The TSTC‐MS was composed of a PDMS microfluidic chip, a flow control system with two syringe pumps (WH‐SSP‐08, Wenhao, China), one heating magnetic stirrer (C‐MAG HS 7, IKA, Germany), two polytetrafluoroethylene tubes (inner diameter = 1.0 mm), and a silicone tube (inner diameter = 2.0 mm). The emulsion was obtained by mixing the water phase (DNP‐sol, GDNP‐sol and LMGDNP‐sol, supplemented with genipin (0.02% w/v)^[^ ^67^ ^]^) and oil phase (mineral oil (Aladin, China) containing 20% v/v span 80 (Damas, Switzerland)) in the microfluidic chip at 4°C, where a water‐in‐oil droplet formed by the shear force. The formation of droplets was visualized using a compact inverted lab microscope (Lux2, CytoSMART, Germany). The flow rate ratio of the water/oil phase was 1:10. Then, the emulsion flowed into the silicone tube that was immersed in a 37°C water bath for gelation to generate the microspheres. Then, for demulsification, microspheres were instilled into a double‐layer liquid bath consisting of diethyl ether and PBS solution to remove mineral oil, obtaining the microspheres in the PBS solution. After being centrifuged and washed with PBS three times, the microspheres were stored at 37°C for further use. For sterilization, microspheres were centrifuged at 2000 rpm for 5 min to remove the supernatant and resuspended in 75% ethanol for 24 h at 25°C. Finally, the microspheres were washed with sterilized PBS three times to remove residual ethanol.

The release of LOX from LMGDNPs was evaluated with a BCA assay. 100 µg LM, 10 mg LMGDNPs or 9.9 mg GDNPs were evenly distributed in 2 mL PBS and placed at 37°C. At 0, 6 h, 12 h and every other day until the 7th day, the supernatant was collected to detect the released protein, while another 2 mL of PBS was added. The difference in detected absorbance between the LMGDNP and GDNP groups resulted from the release of LOX.

The loading capacity of MnO_2_ on LMGDNPs were evaluated by inductively coupled plasma mass spectrometry (ICP‐MS). The loading efficiency % = M_1_/M_0_ × 100% (M_0_: initial MnO_2_ content; M_1_: MnO_2_ content in LMGDNP).

The release of glucose from LMGDNPs and GDNPs were evaluated with a Glucose (Glu) Colorimetric Assay Kit (GOD‐POD Method) (Elabscience, China). Ten milligrams of LMGDNPs and 10 mg of GDNPs were evenly distributed in 500 µL of PBS and placed at 37°C. At 0, 6 h, 12 h and every other 2 days until the 28th day, the supernatant was collected to detect the released glucose, while another 500 µL of PBS was added to resuspend LMGDNPs.

To identify the micromorphology change of LMGDNPs during degradation, 10 mg LMGDNPs were immersed in 1 mL PBS solution and agitated in a shaker at 37°C for 2 weeks. After 0, 3, 7, and 14 days, LMGDNPs were dried with supercritical carbon dioxide, respectively. The diameters were assessed by analyzing the SEM images of LMGDNPs.

The capacity of LM to consume lactate was evaluated using a lactic acid content detection kit (HYKC023, HYCEZMBIO, China). LMGDNPs and GDNPs (1 mg mL^−1^) were added to 500 µL of lactate solution (6 mm). The amount of LM added to the lactate solution was 10 µg mL^−1^ to ensure that the amount of LOX and MnO_2_ nanoparticles was the same as that in LMGDNPs. The reaction system was shaken and placed at 37°C. Then, the concentration of lactate was determined at 0, 15, 30, 60, 120, 180, 240, and 480 min.

Similarly, LMGDNPs, GDNPs, and LM nanoparticles were added to H_2_O_2_ solution (3%). The consumption of H_2_O_2_ was determined by detecting the production of oxygen with a dissolved oxygen meter (REX, JPSJ‐605F, China).

### Scanning Electron Microscopy (SEM)

Microspheres were dehydrated in a sequentially graded ethanol series (25, 50, 75, and 100%) for 15 min in each grade and carefully dried using a supercritical carbon dioxide dryer (EM CPD300, Leica, Germany). Processed microspheres were attached to conductive tape on a standard sample stand and sputter‐coated with gold for 10 min (E‐1045, HITACHI, Japan). The surface morphology was characterized using SEM (Regulus 8230, HITACHI, Japan).

### X‐Ray Photoelectron Spectroscopy (XPS)

XPS (Thermo Scientific K‐Alpha XPS System) was applied to analyze the chemical characteristics of LM nanozyme at binding energies ranging from 620 to 670 eV. The specimens were casted by dropping the nanoparticles on a glass slide and kept for drying overnight in a vacuum dryer before analysis.^[^ ^68^ ^]^

### Isolation, Culture, and Characteristics of BMSCs and NPCs

All procedures involving human tissues were approved by the Medical Ethics Committee of Tongji Medical College, Huazhong University of Science and Technology, China, and followed the Code of Ethics of the World Medical Association (Declaration of Helsinki). Informed consent was obtained from all participating subjects. The information about the BMSCs donors was listed in Supplementary table 1. As previously described, BMSCs were extracted from the bone marrow in artificial hip joint replacements and cultured in complete medium for human mesenchymal stem cells (Cyagen, California, USA).^[^ ^24^ ^]^ Cells were characterized by means of cellular markers (anti‐CD73‐PE, anti‐CD44‐APC, anti‐CD90‐FITC, anti‐CD34‐FITC, Biolegend, San Diego, USA) (Figure S2A, Supporting Information) and osteogenic, chondrogenic and adipogenic differentiation kits (Figure S2B, Supporting Information).

NP tissue was separated from patients with lumbar disc herniation through a microscope operation. NPCs (NPCs) were collected and cultured as previously described.^[^ ^69^ ^]^ Briefly, NP tissue was cut into small pieces and immersed in 0.02% type II collagenase for 2 h at 37 °C to remove the extracellular components. Then, the suspension was centrifuged at 300 × g for 5 min to collect NPCs. Subsequently, these primary cells were cultured with Dulbecco's modified Eagle's medium (DMEM) supplemented with 10% fetal bovine serum (FBS) (Gibco, California, USA) and 1% penicillin/streptomycin (Sigma, St. Louis, MO, USA). The culture medium was changed every 3 days.

Cells were digested using 0.25% trypsin‐0.02% ethylenediaminetetraacetic acid (EDTA, Sigma, Missouri, USA) when the confluence reached 80–90% and passaged for expansion. The third generation was used throughout the following experiments.

### BMSCs Culture on the Microspheres

Gelatin methacryloyl microspheres (GelMA) were purchased from EFL‐Tech Co., Ltd, Suzhou, China, and served as a control group for biocompatibility evaluation. BMSCs suspensions with or without sterilized microspheres were placed in ultralow attachment 24‐well plates (3473, Corning, New York, USA) with ≈1 × 10^5^ cells and 1000 microspheres in each well, forming cell‐loaded microspheres and cell pellets, which were cultured in complete medium for human mesenchymal stem cells at 37°C with 5% CO_2_. Twenty‐four hours later, the medium was changed for further experiments.

For biocompatibility evaluation, the medium was changed to DMEM/F12 culture medium (Gibco) supplemented with 10% FBS (Gibco) and 1% penicillin/streptomycin (Sigma) every 3 days. Cell counting kit‐8 assays (CCK8 assay, Dojindo, Japan) were performed on Days 0, 7, 14, and 21 according to the manufacturer's protocol. Calcein‐AM/propidium iodide staining (Beyotime, China) was used to label the live and dead cells on the 21st day.

For differentiation evaluation, the medium was changed to DMEM/F12 culture medium (Gibco) supplemented with 1% penicillin/streptomycin (Sigma) every 3 days. The cell‐loaded microspheres were collected on the 14th and 21st days for RT‒PCR and immunoreactive staining with the cellular markers of NPCs.

### Evaluation of the Effects of LMGDNPs Against Lactate

BMSCs‐loaded microspheres were cultured in complete medium for human mesenchymal stem cells with lactate (6 mM, HY‐Y0479, MCE, New Jersey, USA) for 24 h. They were collected for CCK8 assay (Dojindo) and Calcein‐AM/propidium iodide staining (Beyotime) evaluation.

NPCs were cocultured with GDNPs (≈4000 microspheres/well for 6‐well plates) or LMGDNPs (≈4000 microspheres per well for 6‐well plates) and incubated with lactate‐rich complete medium for 24 h. Then, cells were collected for the evaluation of cell viability using the CCK8 assay (Dojindo) and apoptosis using western blot, flow cytometry and TUNEL assays.

### Lentivirus Transfection

Autophagic flux was evaluated using AdV‐mRFP‐GFP‐LC3 (HB‐AP20102304, Hanbio, Shanghai, China). Specifically, the colocalization of GFP and RFP fluorescence indicates an autophagosome, while the RFP punctum without GFP indicates an autolysosome. NPCs were transfected with AdV‐mRFP‐GFP‐LC3 at a multiplicity of infection (MOI) of 50 for 8 hours, and then the medium was changed to complete medium for NPCs. Twenty‐four hours later, the cells were cocultured with lactate, LM, and microspheres (≈1000 microspheres per well for 24‐well plates) for another 24 h. Then, images were captured by confocal microscopy (Olympus, Japan). The number of puncta per cell was quantified based on multiple cells from three independent experiments.

The shRNA against human transforming growth factor β2 overlapping transcript 1 (TGFB2‐OT1) was synthesized and subcloned into a lentivirus (Shanghai Genechem Co., Ltd., Shanghai, China), obtaining shTGFB2‐OT1. NPCs were plated in 6‐well plates (≈1.5 × 10^4^ cells per well). After 24 hours, cells were transfected with shTGFB2‐OT1 and negative control at an MOI of 100 for 12 h. Then, the medium was changed to complete medium for NPCs. After 72 h, the knockdown efficiency was determined using the protein expression level of TGFB2‐OT1. Furthermore, the transfected cells were expanded for further experiments.

### Apoptosis Assay

Treated NPCs were collected by trypsinization and centrifugation, washed with ice‐cold PBS and resuspended in 100 µL of 1x binding buffer. Two microliters of propidium iodide (PI) and 2 µL of Annexin‐V (HYC2019, HYCEZMBIO, China) were then added. Cells were incubated in the dark at room temperature for 10 min and detected using flow cytometry (BD LSRII, Becton Dickinson) within 30 min. Annexin+/PI+ (indicating late apoptosis) and Annexin+/PI‐ (indicating early apoptosis) cells were used to calculate the apoptotic rate.

TUNEL assays of NPCs or histological sections were performed using the One Step TUNEL Apoptosis Assay Kit (HY88576, HYCEZMBIO, Wuhan, China). Briefly, cells were permeabilized with 0.1% w/v Triton X‐100 and 0.1% w/v sodium citrate for 15 min at 25°C and were incubated with 100 µL of TUNEL reaction mixture for 60 min at 37°C in the dark. Next, the nucleus was stained with 4′,6‐diamidino‐2‐phenylindole (DAPI, Beyotime, China) for 10 min before images were captured using a fluorescence microscope (Olympus, Japan).

### Real‐Time PCR

Total RNA was extracted using the RNA‐easyTM Isolation Reagent (Vazyme Biotech Co., Ltd, Nanjing, China). The concentration and purity of the extracted RNA were assessed using a NanoDrop 2000 (Thermo Fisher Scientific, Massachusetts, USA). A TaqMan microRNA assay kit (Applied Biosystems, Foster City, USA) and reverse transcription kit (Vazyme Biotech Co., Ltd, Nanjing, China) were used for cDNA synthesis of microRNA and total mRNA, respectively. Gene expression was detected using the SYBR PrimeScript RT‒PCR Kit (Vazyme Biotech Co., Ltd, Nanjing, China) on a Step One Plus Real‐Time PCR system (Bio‐Rad, Hercules, CA, USA). The fold changes were determined using the 2 ‐△△CT method with GAPDH and U6 for normalization. The primer sequences used are listed in Table S2 (Supporting Information).

### Western Blot

After removing the medium, NPCs were washed with PBS three times and lysed in lysis buffer (Beyotime, Jiangsu, China) with the protease inhibitor phenylmethanesulfonyl fluoride (PMSF, Beyotime) and phosphatase inhibitor cocktail I (Sigma, USA). Cells were disrupted using an ultrasonic cell disruptor (Sonics & Materials, Inc., USA). The lysates were then centrifuged at 15000 × *g* for 15 min to collect the supernatants. The protein concentration was determined using a BCA protein assay kit (Boster, China). After being denatured using SDS‒PAGE denaturation buffer (Servicebio, China), the supernatants were loaded onto 10% or 15% SDS‒PAGE gels. Western blotting was performed as previously described.^[^ ^70^ ^]^ The following antibodies were used: Collagen Type II (Col2, 1:1000, A1560, Abclonal, China), Aggrecan (Acan, 1:1000, A11691, Abclonal, China), Sox9 (1:1000, ab185966, Abcam, UK), MMP3 (1:1000, A11418, ABclonal, China), MMP13 (1:1000, A11755, ABclonal, China), Adamts5 (1:1000, A2836, Abclonal, China), Bcl2 (1:1000, 26593‐1‐AP, Proteintech, USA), Bax (1:1000, 50599‐2‐lg, Proteintech, USA), caspase3 (1:1000, 19677‐1‐AP, Proteintech, USA), LC3 (1:1000, 12741T, CST, USA) and GAPDH (1:3000, ab181602, Abcam, UK).

### Bioinformatics Analysis

The generation of the RNA‐seq data in this study has been previously described. Briefly, 2.5 µg of total RNA from each sample was sequenced using an Illumina platform (Illumina, Inc., San Diego, CA, USA) and a HiSeq 2000 sequencing system. After quality control, the reads in each sample were mapped to the human genome and counted. The expression level of each gene was calculated as the normalized count value. The differentially expressed genes were extracted with |log_2_FC| > 1 and FDR‐adjusted p value < 0.05 using DEseq2.

### IDD Model and Cell‐Loaded Microsphere Injection

With the ethical approval of the Institutional Animal Care and Use Committee, Huazhong University of Science and Technology ([2022] IACUC Number 3180), Sprague Dawley (SD) male rats of competent immune status were grouped as Blank, Lactate + CTRL, Lactate + cell pellets (CP), Lactate + GDNP, and Lactate + LMGDNP. Two percent (w/v) pentobarbital (40 mg kg^−1^) was used to anesthetize SD male rats. Then, the coccygeal vertebrae Co5/6 were recognized using manual palpation and confirmed using radiography. For the blank group, normal saline (5 µL) was injected into the operated disc using a 29‐gauge needle. For other groups, IDD models were established by injecting 5 µL lactate solution (6 mM) via a 29‐gauge needle. BMSCs (1×10^5^) with or without 1000 microspheres were placed in an ultralow attachment 24‐well plate (Corning) and cultured in complete medium for human mesenchymal stem cells at 37°C with 5% CO_2_ for 24 h to form CP or BMSCs‐loaded microspheres. Then, normal saline (5 µL for the lactate group), CP (≈150 particles, 5 µL) or cell‐loaded microspheres (≈150 particles, 5 µL) were injected into the operated disc using a 29‐gauge needle at the first week after lactate injection. Lactate solution was reinjected at the 4th week. CP and cell‐loaded microspheres (≈150 particles, 5 µL) were reinjected at the 5th week. Radiological and histological analyses were performed at the 4th and 8th weeks after the first dose of lactate injection.

### BMSCs Intradisc Survival Evaluation

SD male rats were obtained for evaluating the survival of implanted BMSCs and grouped as lactate + CTRL, lactate + cell pellets (CP), lactate + GDNP, and lactate + LMGDNP. BMSCs were stained with 1,1‐dioctadecyl‐3,3,3,3‐tetramethyl indotricarbocyanine iodide (DiR iodide, 10 µg mL^−1^; AAT‐22070, AAT Bioquest, China) as previously described^[^ ^71^ ^]^ before they were planted to form CP‐ or BMSCs‐loaded microspheres. Then, labeled cell pellets and cell‐loaded microspheres were injected into the discs following the same protocol described above. On the 1st day, 7th day, 14th day and 21st day, BMSCs stained with DiR were tracked using IVIS Lumina Imaging Systems (Xenogen, GEORGIA, USA). The disc samples at different time points were collected for histological staining, which was visualized by a fluorescence microscope (Olympus, Tokyo, Japan).

### Magnetic Resonance Imaging (MRI)

The coronal plane of discs was scanned using a 3.0 T MRI scanner (GE Medical Systems, UK) to obtain the T2‐weighted midsagittal sections. Three spine surgeons graded the discs independently, following the Pfirrmann MRI‐grade system. Then, the T2‐weighted signaling intensity in the NP area, indicating the hydration of disc tissues, was measured by ImageJ (National Institutes of Health, Bethesda, MD). Relative water content was calculated as follows: relative water content = $\frac{D_{s}}{D_{0}}$ (D_s_, T2‐weighted intensity of discs after surgery; D_o_, T2‐weighted intensity of intact discs).

### Histological Evaluation

Disc tissues were fixed in paraformaldehyde (4% w/v) and decalcified in formic acid solution (10% by wt.) for 4 weeks. Then, discs were embedded in paraffin and cut into 4.0 µm sections using a rotary microtome (Thermo Scientific, USA). The slides were stained with hematoxylin and eosin (HE) and Safranin O‐fast green (SO) according to the manufacturer's instructions. Three independent researchers evaluated the degenerative degree according to the Han et al. histological grading scale.^[^ ^72^ ^]^

### Immunofluorescence Staining

Immunofluorescence staining was performed using primary antibodies against CD24 (1:150, ab202073, Abcam, UK), Krt19 (1:100, A19040, Abclonal, China), Col2 (1:100, A1560, Abclonal, China), Acan (1:150, A11691, Abclonal, China), MMP3 (1:100, ab52915, Abcam, UK), and Sox9 (1:100, ab185966, Abcam, UK). Fluorescence‐conjugated secondary antibodies (Invitrogen, USA) were used to visualize the fluorescent signals before the slides were stained with DAPI (Beyotime, USA). The slides were sealed using an anti‐fluorescence quenching sealer (Thermo Fisher Scientific, Massachusetts, USA). Then, fluorescence signals were captured using a fluorescence microscope (Olympus, Tokyo, Japan).

### Statistical Analyses

Each experiment was performed with at least three biological replicates (n). Accordingly, data are presented as the mean ± standard deviation (SD) and analyzed using SPSS 19. Differences between the two groups were analyzed using Student's t test. One‐way repeated‐measures analysis of variance (ANOVA) and least significant difference (LSD) were used to analyze multiple datasets. All statistical charts were drawn using GraphPad Prism 7 software (GraphPad Software Inc., San Diego, CA, USA).

## Conflict of Interest

The authors declare no conflict of interest.

## Author Contributions

Y.P., X.C., and Q.Z. contributed equally to this work and share first authorship. Y.P. performed conceptualization, methodology, software, validation, formal analysis, investigation, resources, data curation, writing‐original draft, visualization, project administration, and funding acquisition. X.C. performed methodology, software, validation, data curation, investigation, and project administration. Q.Z. performed methodology, software, validation, resources, and data curation. S.L. performed software, validation, formal analysis, and data curation. W.W. performed project administration and writing‐review & editing. K.L. performed methodology, validation, formal analysis, and data curation. H.L. performed methodology, validation, formal analysis, investigation, and funding acquisition. X.Q. performed methodology, writing‐review & editing, and funding acquisition. Y.X. performed methodology, software, validation, resources. B.W. performed writing‐review & editing, supervision, and funding acquisition. D.Q. performed conceptualization, writing‐review & editing. S.F. performed writing‐review & editing. Z.R. performed methodology, conceptualization, supervision, writing‐review & editing. Y.B. performed conceptualization, supervision, and writing‐review & editing. Z.S. performed conceptualization, resources, writing‐review & editing, supervision, and funding acquisition.

## Supporting information

Supporting Information

## Data Availability

The data that support the findings of this study are available from the corresponding author upon reasonable request.

## References

[1] A. Cieza , K. Causey , K. Kamenov , S. W. Hanson , S. Chatterji , T. Vos , Lancet 2021, 396, 2006. 33275908 10.1016/S0140-6736(20)32340-0 PMC7811204

[2] W. Brinjikji , F. E. Diehn , J. G. Jarvik , C. M. Carr , D. F. Kallmes , M. H. Murad , P. H. Luetmer , Am. J. Neuroradiol. 2015, 36, 2394. 26359154 10.3174/ajnr.A4498 PMC7964277

[3] S. Ohtori , G. Inoue , M. Miyagi , K. Takahashi , Spine J 2015, 15, 1347. 24657737 10.1016/j.spinee.2013.07.490

[4] J. Guevar , N. Olby , Minimally invasive microsurgical decompression of an intervertebral disc protrusion in a dog., United States, 2020, Vol. 49 Suppl 1, pp. O86–O92. 10.1111/vsu.13263 31237005

[5] F. H. Geisler , P. C. Mcafee , R. J. Banco , S. L. Blumenthal , R. D. Guyer , R. T. Holt , M. E. Majd , SAS J 2009, 3, 17. 25802625 10.1016/SASJ-2008-0019-RR PMC4365588

[6] S. Zou , J. Gao , B. Xu , X. Lu , Y. Han , H. Meng , Eur. spine J. Off. Publ. Eur. Spine Soc. Eur. Spinal Deform. Soc. Eur. Sect. Cerv. Spine Res. Soc. 2017, 26, 985. 10.1007/s00586-016-4655-5 27314663

[7] S. M. Richardson , G. Kalamegam , P. N. Pushparaj , C. Matta , A. Memic , A. Khademhosseini , R. Mobasheri , F. L. Poletti , J. A. Hoyland , A. Mobasheri , Methods 2016, 99, 69. 26384579 10.1016/j.ymeth.2015.09.015

[8] N. Herger , P. Bermudez‐Lekerika , M. Farshad , C. E. Albers , O. Distler , B. Gantenbein , S. Dudli , 2022, 23, 2721. 10.3390/ijms23052721 PMC8910866 35269863

[9] Y. Peng , J. Li , H. Lin , S. Tian , S. Liu , F. Pu , L. Zhao , K. Ma , X. Qing , Z. Shao , Biomater. Transl. 2021, 2, 343. 35837417 10.12336/biomatertransl.2021.04.008 PMC9255795

[10] J. Clouet , M. Fusellier , A. Camus , C. Le Visage , J. Guicheux , Adv. Drug Delivery Rev. 2019, 146, 306. 10.1016/j.addr.2018.04.017 29705378

[11] S. Motaghinasab , A. Shirazi‐Adl , M. Parnianpour , J. P. G. Urban , Eur. spine J. Off. Publ. Eur. Spine Soc. Eur. Spinal Deform. Soc. Eur. Sect. Cerv. Spine Res. Soc. 2014, 23, 715. 10.1007/s00586-013-3142-5 PMC3960428 24375329

[12] S. Rajasekaran , K. Venkatadass , J. Naresh Babu , K. Ganesh , A. P. Shetty , Eur. spine J. Off. Publ. Eur. Spine Soc. Eur. Spinal Deform. Soc. Eur. Sect. Cerv. Spine Res. Soc. 2008, 17, 626. 10.1007/s00586-008-0645-6 PMC2367412 18357472

[13] M. Loibl , K. Wuertz‐Kozak , G. Vadala , S. Lang , J. Fairbank , J. P. Urban , JOR spine 2019, 2, e1043. 31463457 10.1002/jsp2.1043 PMC6711491

[14] A. Moya , N. Larochette , J. Paquet , M. Deschepper , M. Bensidhoum , V. Izzo , G. Kroemer , H. Petite , D. Logeart‐Avramoglou , Stem Cells 2017, 35, 181. 27578059 10.1002/stem.2493

[15] E. M. Bartels , J. C. T. Fairbank , C. P. Winlove , J. P. G. Urban , Spine (Phila. Pa. 1976). 1998, 23, 1. 9460145 10.1097/00007632-199801010-00001

[16] W. Wu , X. Zhang , X. Hu , X. Wang , L. Sun , X. Zheng , L. Jiang , X. Ni , C. Xu , N. Tian , S. Zhu , H. Xu , J. Orthop. Res. Off. Publ. Orthop. Res. Soc. 2014, 32, 253. 10.1002/jor.22503 24307209

[17] K. Wuertz , K. Godburn , J. C. Iatridis , Biochem. Biophys. Res. Commun. 2009, 379, 824. 19133233 10.1016/j.bbrc.2008.12.145 PMC2652844

[18] C. Xia , Z. Zeng , B. Fang , M. Tao , C. Gu , L. Zheng , Y. Wang , Y. Shi , C. Fang , S. Mei , Q. Chen , J. Zhao , X. Lin , S. Fan , Y. Jin , P. Chen , Biol. Med. 2019, 143, 1. 10.1016/j.freeradbiomed.2019.07.026 31351174

[19] F. Alam , S. Roychoudhury , A. H. Jalal , Y. Umasankar , S. Forouzanfar , N. Akter , S. Bhansali , N. Pala , Biosens. Bioelectron. 2018, 117, 818. 30096736 10.1016/j.bios.2018.06.054

[20] J. Shen , A. Chen , Z. Cai , Z. Chen , R. Cao , Z. Liu , Y. Li , J. Hao , Bioact. Mater. 2022, 12, 153. 35310385 10.1016/j.bioactmat.2021.10.013 PMC8897073

[21] S. A. Ansari , Q. Husain , Biotechnol. Adv. 2012, 30, 512. 21963605 10.1016/j.biotechadv.2011.09.005

[22] M. Fiordalisi , A. J. Silva , M. Barbosa , R. Gonçalves , J. Caldeira , Trends Biotechnol. 2020, 38, 947. 32466967 10.1016/j.tibtech.2020.05.002

[23] Z. Lin , Z. Rao , J. Chen , H. Chu , J. Zhou , L. Yang , D. Quan , Y. Bai , ACS Biomater. Sci. Eng. 2022, 8, 1644. 35357124 10.1021/acsbiomaterials.1c01474

[24] Y. Peng , X. Qing , H. Lin , D. Huang , J. Li , S. Tian , S. Liu , X. Lv , K. Ma , R. Li , Z. Rao , Y. Bai , S. Chen , M. Lei , D. Quan , Z. Shao , Bioact. Mater. 2021, 6, 3541. 33842740 10.1016/j.bioactmat.2021.03.014 PMC8022111

[25] S. F. Badylak , Ann. Biomed. Eng. 2014, 42, 1517. 24402648 10.1007/s10439-013-0963-7

[26] H. T. Norbertczak , E. Ingham , H. L. Fermor , R. K. Wilcox , Tissue Eng., Part C 2020, 26, 565. 10.1089/ten.tec.2020.0104 PMC7698987 33050844

[27] Z. Chen , C. Wang , N. Yu , L. Si , L. Zhu , A. Zeng , Z. Liu , X. Wang , Biomed. Pharmacother. 2019, 111, 151. 30579254 10.1016/j.biopha.2018.12.046

[28] K. Zhao , R. An , Q. Xiang , G. Li , K. Wang , Y.u Song , Z. Liao , S. Li , W. Hua , X. Feng , X. Wu , Y. Zhang , A. Das , C. Yang , Cell Prolif 2021, 54, e12941. 33111436 10.1111/cpr.12941 PMC7791185

[29] R. He , Z. Wang , M. Cui , S. Liu , W. Wu , M. Chen , Y. Wu , Y. Qu , H. Lin , S. Chen , B. Wang , Z. Shao , Autophagy 2021, 11, 3338. 10.1080/15548627.2021.1872227 PMC8632345 33455530

[30] V. Madhu , A. R. Guntur , M. V. Risbud , Matrix Biol. 2021, 100–101, 207. 10.1016/j.matbio.2020.12.002 PMC8180533 33301899

[31] L. Luo , X. Jian , H. Sun , J. Qin , Y. Wang , J.i Zhang , Z. Shen , D.i Yang , C. Li , P. Zhao , M. Liu , Z. Tian , Y. Zhou , Stem Cells 2021, 39, 467. 33459443 10.1002/stem.3322 PMC8048856

[32] S. Huang , W. Lu , D.i Ge , N. Meng , Y. Li , L.e Su , S. Zhang , Y. Zhang , B. Zhao , J. Miao , Autophagy 2015, 11, 2172. 26565952 10.1080/15548627.2015.1106663 PMC4835209

[33] A. R. Pessentheiner , H. J. Pelzmann , E. Walenta , M. Schweiger , L. N. Groschner , W. F. Graier , D. Kolb , K. Uno , T. Miyazaki , A. Nitta , D. Rieder , A. Prokesch , J. G. Bogner‐Strauss , J. Biol. Chem. 2013, 288, 36040. 24155240 10.1074/jbc.M113.491324 PMC3861652

[34] W. Jiang , B. Ogretmen , Autophagy 2013, 9, 258. 23182807 10.4161/auto.22739 PMC3552895

[35] S. A. Turner , K. T. Wright , P. N. Jones , B. Balain , S. Roberts , Stem Cells Int 2016, 2016, 5415901. 26977156 10.1155/2016/5415901 PMC4764757

[36] S. M. Naqvi , C. T. Buckley , Spine (Phila. Pa. 1976). 2016, 41, 743. 26630431 10.1097/BRS.0000000000001314

[37] X. Zhang , X. Chen , H. Hong , R. Hu , J. Liu , C. Liu , Bioact. Mater. 2022, 10, 15. 34901526 10.1016/j.bioactmat.2021.09.014 PMC8637010

[38] Y. Peng , X. Chen , Z. Rao , W. Wu , H. Zuo , K. Chen , K. Li , H. Lin , S. Liu , Y. Xiao , B. Wang , D. Quan , X. Qing , Y. Bai , Z. Shao , Acta Biomater. 2023, 170, 288. 37598791 10.1016/j.actbio.2023.08.028

[39] T. J. Keane , R. Londono , N. J. Turner , S. F. Badylak , Biomaterials 2012, 33, 1771. 22137126 10.1016/j.biomaterials.2011.10.054

[40] G. G. Giobbe , C. Crowley , C. Luni , S. Campinoti , M. Khedr , K. Kretzschmar , M. M. De Santis , E. Zambaiti , F. Michielin , L. Meran , Q. Hu , G. Van Son , L. Urbani , A. Manfredi , M. Giomo , S. Eaton , D. Cacchiarelli , V. S. W. Li , H. Clevers , P. Bonfanti , N. Elvassore , P. De Coppi , Nat. Commun. 2019, 10, 5658. 31827102 10.1038/s41467-019-13605-4 PMC6906306

[41] M. T. Wolf , K. A. Daly , E. P. Brennan‐Pierce , S. A. Johnson , C. A. Carruthers , A. D'amore , S. P. Nagarkar , S. S. Velankar , S. F. Badylak , Biomaterials 2012, 33, 7028. 22789723 10.1016/j.biomaterials.2012.06.051 PMC3408574

[42] Y. Xu , J. Zhou , C. Liu , S. Zhang , F. Gao , W. Guo , X. Sun , C. Zhang , H. Li , Z. Rao , S. Qiu , Q. Zhu , X. Liu , X. Guo , Z. Shao , Y. Bai , X. Zhang , D. Quan , Biomaterials 2021, 268, 120596. 33341040 10.1016/j.biomaterials.2020.120596

[43] P. Qiu , M. Li , K. Chen , B. Fang , P. Chen , Z. Tang , X. Lin , S. Fan , Biomaterials 2020, 227, 119552. 31670079 10.1016/j.biomaterials.2019.119552

[44] A. Nishiguchi , T. Taguchi , Acta Biomater. 2021, 131, 211. 34198010 10.1016/j.actbio.2021.06.033

[45] Y. Lei , Y. Wang , J. Shen , Z. Cai , C. Zhao , H. Chen , X. Luo , N. Hu , W. Cui , W. Huang , Sci. Adv. 2022, 8, eabl6449. 35108047 10.1126/sciadv.abl6449 PMC8809544

[46] T. Guehring , G. Wilde , M. Sumner , T. Grünhagen , G. B. Karney , U. K. Tirlapur , J. P. G. Urban , Arthritis Rheum. 2009, 60, 1026. 19333932 10.1002/art.24407

[47] X. Yin , A. Motorwala , O. Vesvoranan , H. B. Levene , W. Gu , C.‐Y. Huang , Sci. Rep. 2020, 10, 8899. 32483367 10.1038/s41598-020-65691-w PMC7264337

[48] C.‐Y. Huang , W. Y. Gu , J. Biomech. 2008, 41, 1184. 18374341 10.1016/j.jbiomech.2008.02.002 PMC2398770

[49] D. Mokhbi Soukane , A. Shirazi‐Adl , J. P. G. Urban , J. Biomech. 2007, 40, 2645. 17336990 10.1016/j.jbiomech.2007.01.003

[50] M. Bez , Z. Zhou , D. Sheyn , W. Tawackoli , J. C. Giaconi , G. Shapiro , S. Ben David , Z. Gazit , G. Pelled , D. Li , D. Gazit , Sci. Rep. 2018, 8, 17363. 30478330 10.1038/s41598-018-34582-6 PMC6255799

[51] B. Diamant , J. Karlsson , A. Nachemson , Experientia 1968, 24, 1195. 5703005 10.1007/BF02146615

[52] B. Levine , G. Kroemer , Cell 2019, 176, 11. 30633901 10.1016/j.cell.2018.09.048 PMC6347410

[53] A. C. Kimmelman , E. White , Cell Metab. 2017, 25, 1037. 28467923 10.1016/j.cmet.2017.04.004 PMC5604466

[54] U. C. Anozie , P. Dalhaimer , Adv. Drug Delivery Rev. 2017, 122, 65. 10.1016/j.addr.2017.01.001 28065863

[55] I. Tanida , Microbiol. Immunol. 2011, 55, 1. 21175768 10.1111/j.1348-0421.2010.00271.x

[56] F. Chen , H. Liu , X. Wang , Z. Li , J. Zhang , Y. Pei , Z. Zheng , J. Wang , Osteoarthr. Cartil. 2020, 28, 1121. 10.1016/j.joca.2020.05.011 32470597

[57] J. Wang , J. Hu , X. Chen , C. Huang , J. Lin , Z. Shao , M. Gu , Y. Wu , N. Tian , W. Gao , Y. Zhou , X. Wang , X. Zhang , FASEB J. Off. Publ. Fed. Am. Soc. Exp. Biol. 2019, 33, 11555. 10.1096/fj.201900703R 31331201

[58] Y. Gao , T. Zhang , Curr. Stem Cell Res. Ther. 2021, 16, 23. 32357821 10.2174/1574888X15666200502000807

[59] L. Popp , L. Segatori , Curr. Opin. Biotechnol. 2015, 36, 129. 26340102 10.1016/j.copbio.2015.08.016

[60] Y. Zhang , N.a Kong , Y. Zhang , W. Yang , F. Yan , Theranostics 2017, 7, 1214. 28435460 10.7150/thno.17252 PMC5399588

[61] O. Zabirnyk , M. Yezhelyev , O. Seleverstov , Autophagy 2007, 3, 278. 17351332 10.4161/auto.3916

[62] D. Guo , Y. Zhao , Y. Zhang , Q. Wang , Z. Huang , Q. Ding , Z. Guo , X. Zhou , L. Zhu , N. Gu , J. Biomed. Nanotechnol. 2014, 10, 669. 24734519 10.1166/jbn.2014.1625

[63] L. Wu , Y. Zhang , C. Zhang , X. Cui , S. Zhai , Y. Liu , C. Li , H. Zhu , G. Qu , G. Jiang , B. Yan , ACS Nano 2014, 8, 2087. 24552177 10.1021/nn500376w PMC5586106

[64] D. Ge , L. Han , S. Huang , N. Peng , P. Wang , Z. Jiang , J. Zhao , L. Su , S. Zhang , Y. Zhang , H. Kung , B. Zhao , J. Miao , Autophagy 2014, 10, 957. 24879147 10.4161/auto.28363 PMC4091179

[65] B. Zhou , J. Liu , R. Kang , D. J. Klionsky , G. Kroemer , D. Tang , Semin. Cancer Biol. 2020, 66, 89. 30880243 10.1016/j.semcancer.2019.03.002

[66] W. Zhu , Z. Dong , T. Fu , J. Liu , Q. Chen , Y. Li , R. Zhu , L. Xu , Z. Liu , Adv. Funct. Mater. 2016, 26, 5490.

[67] Y. Peng , D. Huang , J. Li , S. Liu , X. Qing , Z. Shao , J. Tissue Eng. Regener. Med. 2020, 14, 497. 10.1002/term.3014 PMC7155128 32012486

[68] G. Lawrie , I. Keen , B. Drew , A. Chandler‐Temple , L. Rintoul , P. Fredericks , L. Grøndahl , Biomacromolecules 2007, 8, 2533. 17591747 10.1021/bm070014y

[69] L. Kang , Q. Xiang , S. Zhan , Y. Song , K. Wang , K. Zhao , S. Li , Z. Shao , C. Yang , Y. Zhang , Oxid. Med. Cell. Longev. 2019, 2019, 7810320. 31976028 10.1155/2019/7810320 PMC6954474

[70] Y. Peng , H. Lin , S. Tian , S. Liu , J. Li , X. Lv , S. Chen , L. Zhao , F. Pu , X. Chen , H. Shu , X. Qing , Z. Shao , Biol. Med. 2021, 177, 247. 10.1016/j.freeradbiomed.2021.10.034 34737144

[71] B. Huang , J. Qian , J. Ma , Z. Huang , Y. Shen , X. Chen , A. Sun , J. Ge , H. Chen , Stem Cell Res. Ther. 2014, 5, 22. 24507665 10.1186/scrt410 PMC4055118

[72] B. Han , K. Zhu , F.‐C. Li , Y.‐X. Xiao , J. Feng , Z.‐L. Shi , M. Lin , J. Wang , Q.‐X. Chen , Spine (Phila. Pa. 1976). 2008, 33, 1925. 18708924 10.1097/BRS.0b013e31817c64a9

