# Supplementary material for: Enzymatically Bioactive Nucleus Pulposus Matrix Hydrogel Microspheres for Exogenous Stem Cells Therapy and Endogenous Repair Strategy to Achieve Disc Regeneration
Source: Adv Sci (Weinh). 2023 Dec 25;11(10):2304761. doi: 10.1002/advs.202304761 (PMC10933624; doi:10.1002/advs.202304761)
Supplement: Supplementary file 1 — Supporting Information [file ADVS-11-2304761-s001.pdf]

ADVANCED SCIENCE
Open Access

Supporting Information

for *Adv. Sci.*, DOI 10.1002/advs.202304761

Enzymatically Bioactive Nucleus Pulposus Matrix Hydrogel Microspheres for Exogenous Stem Cells Therapy and Endogenous Repair Strategy to Achieve Disc Regeneration

Yizhong Peng, Xuanzuo Chen, Qimin Zhang, Sheng Liu, Wei Wu, Kanglu Li, Hui Lin, Xiangcheng Qing, Yan Xiao, BaiChuan Wang, Daping Quan, Shiqing Feng, Zilong Rao, Ying Bai* and Zengwu Shao**

Supplementary Table 1 The information of BMSC donors

Case No.	Sample	Age	Gender	Dignosis	Surgery	Experiments
1	bone marrow	30	Female	Femoral Fracture	Open reduction and internal fixation of lower limb fractures	Multidirectional differentiation potential evaluation, Stem cells markers identification, biocompatibility evaluation, in vitro immunofluorescence staining, PCR assay, animal experiments.
2	bone marrow	19	Male	Femoral Fracture	Open reduction and internal fixation of lower limb fractures	Multidirectional differentiation potential evaluation, Stem cells markers identification, biocompatibility evaluation, in vitro immunofluorescence staining , PCR assay, animal experiments.
3	bone marrow	25	Male	Femoral Fracture	Open reduction and internal fixation of lower limb fractures	Multidirectional differentiation potential evaluation, Stem cells markers identification, biocompatibility evaluation, in vitro immunofluorescence staining, PCR assay, animal experiments.

Supplementary Table 2 The sequences of primers

Target	Sequence (5'-3')	
CD24	Forward primer	TCTCGGCTAATCTCCAAACGCT
	Reverse primer	GCTTCCAGTCCTCACATCCCAA
Krt19	Forward primer	CTGTCCACACTACGCAGATCCA
	Reverse primer	TGTGTCAGCACGCACGTTACTC
Acan	Forward primer	AAGCCCTTGTCTGAATGGAGCC
	Reverse primer	GGTCGGGAAAGTGGCGATAACA
Col2	Forward primer	ACCTCACGCCTTCCCATTGTT
	Reverse primer	TGGGTCCTTTGGGTTCGCAAT
TGFB2-OT1	Forward primer	GCAGTTTCACCTAAAGAGCAGC
	Reverse primer	TTCCTTCCCACCTCCACCC
GAPDH	Forward primer	CAACCGGGAAGGAAATGAATGGG
	Reverse primer	GCCCAATACGACCAAATCAGAGAA
miRNA 4488	RT	GTCGTATCCAGTGCAGGGTCCGAGGTAT TCGCA CTGGATACGACGCCGGAG
	Forward primer	GGTCTCCAGCCTGCCCTTCA
	Reverse primer	GCATCCACGCCGCTCCAT
miRNA 4459	RT	GTCGTATCCAGTGCAGGGTCCGAGGTAT TCGCACTGGATACGACTCCACCT
	Forward primer	ATTATCCAGGAGGCGGAGG
	Reverse primer	TCGTATCCAGTGCAGGGTC
miRNA 3960	RT	GTCGTATCCAGTGCAGGGTCCGAGGTAT TCGCACTGGATACGACCCCCGCC
	Forward primer	CGGCTTCCCCTACAGATG

	Reverse primer	CCACAGCGAATGGACAGA
U6	Forward primer	CTCGCTTCGGCAGCACATA
	Reverse primer	CGAATTTGCGTGTCATCCT

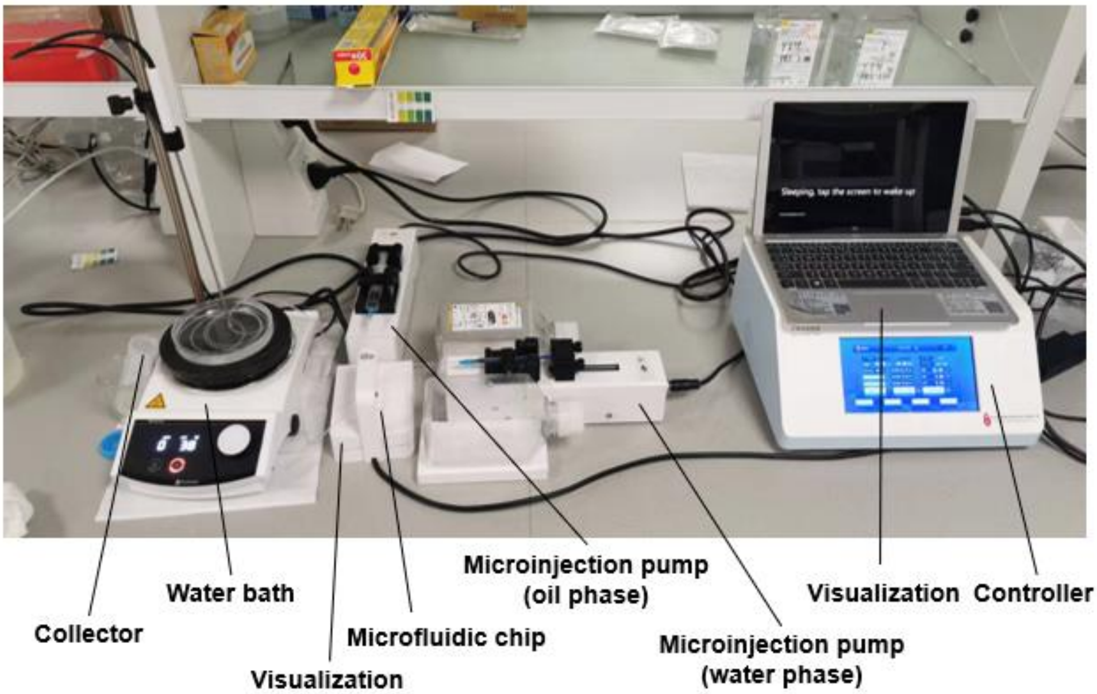

Figure S1. The two-stage temperature-controlling microfluidic system (TSTC-MS).

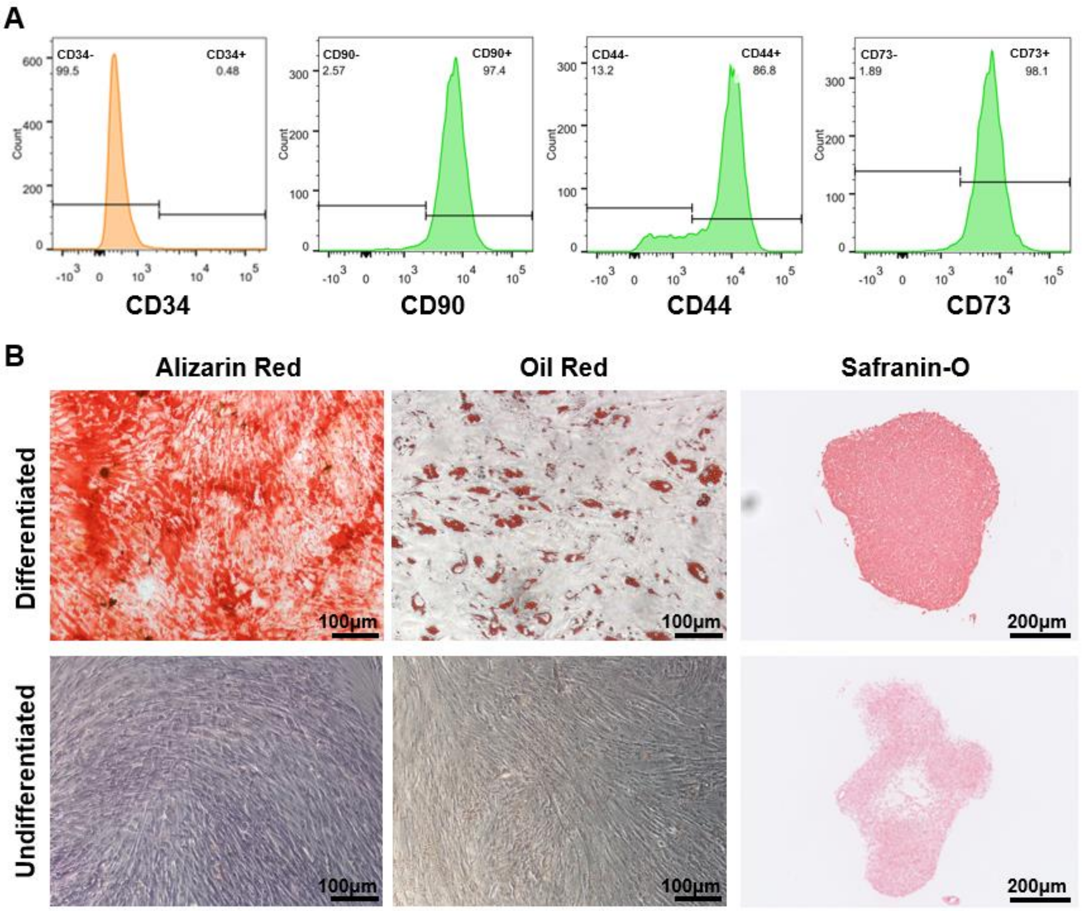

Figure S2. Characteristics of BMSCs. A) Flow cytometric analysis for cell surface markers identification. B) The multi-lineage differentiation potency of MSCs. Alizarin red staining, Oil red O staining and Safranin-O staining confirmed the ability of MSCs to differentiate into osteogenic, chondrogenic and adipogenic lineages, respectively. Scale bar, 100 μm.

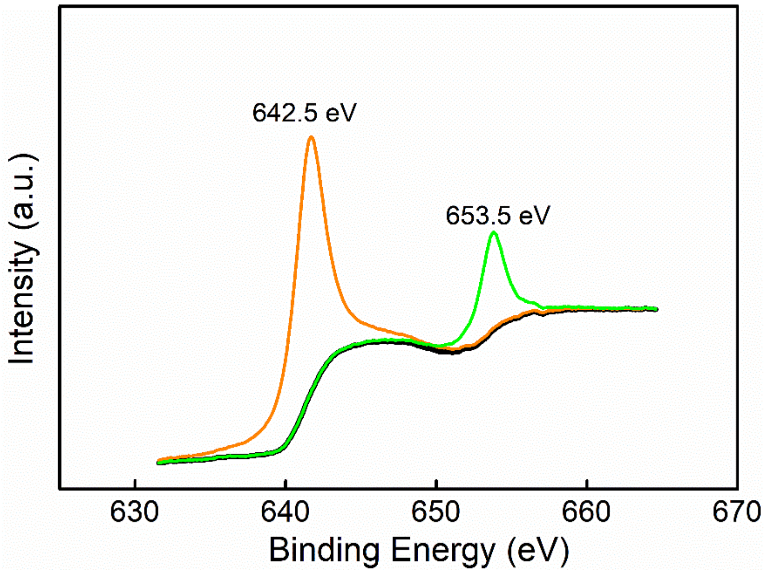

Figure S3. X-ray photoelectron spectroscopy (XPS) of LOX-MnO_2 nanozyme.

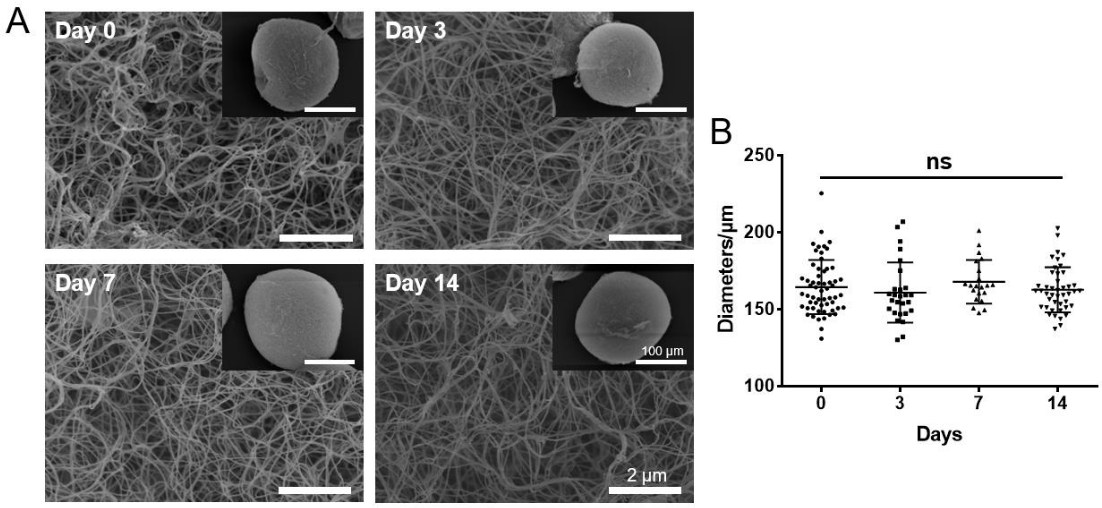

Figure S4. Degradation and morphology change of the microspheres in PBS solution. (A) Representative SEM images of the microspheres and their nanofibrous structures on day 0, 3, 7, and 14. (B) Diameter of the microspheres during degradation process, n>20. ns, no significance.

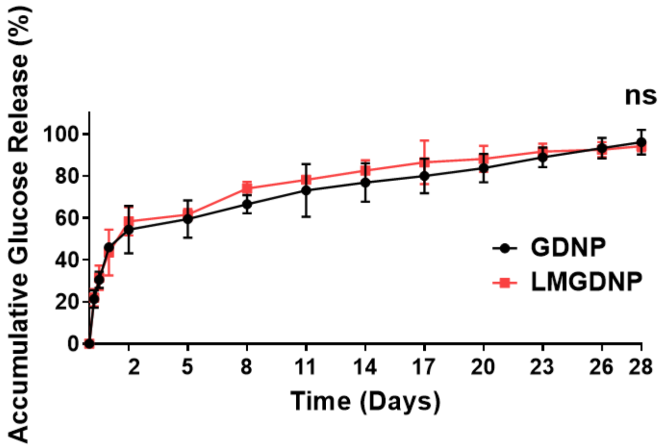

Figure S5. Cumulative release profile of glucose in GDNP and LMGDNPs. Data are presented as the mean ± SD, n = 3, ns, no significance.

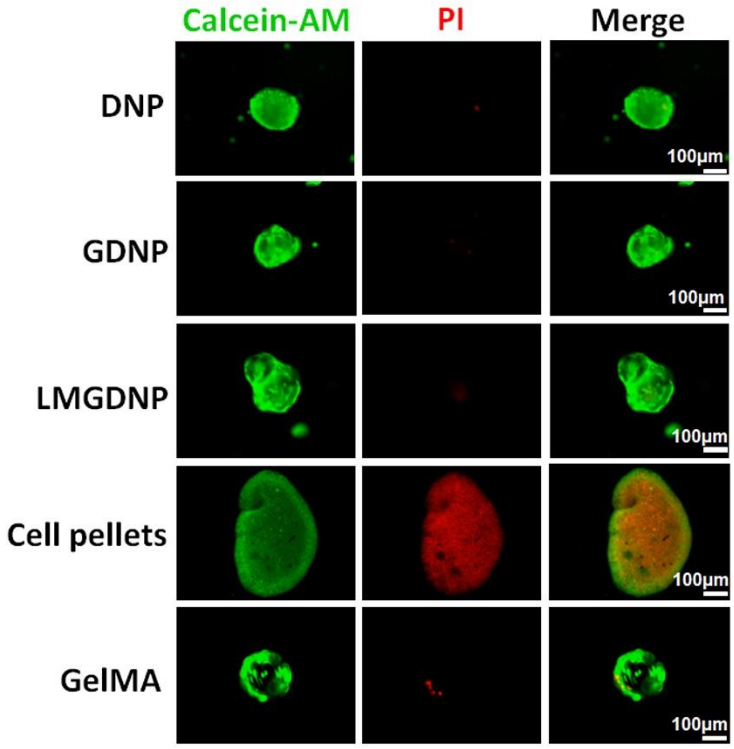

Figure S6. Calcein-AM/PI staining images of the live/dead assays of BMSCs cultured on microspheres (DNP, GDNP, LMGDNP, and GelMA) or cultured as cell pellets for 14 days. Scale bar, 100 μm.

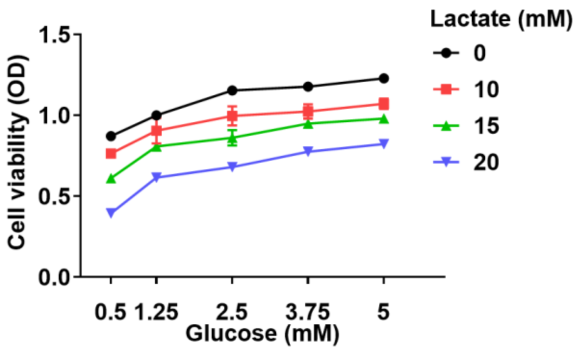

Figure S7. Effects of glucose on cell viability. The viability of BMSCs treated with glucose and lactate with different concentrations. Data are presented as the mean ± SD, n = 3.

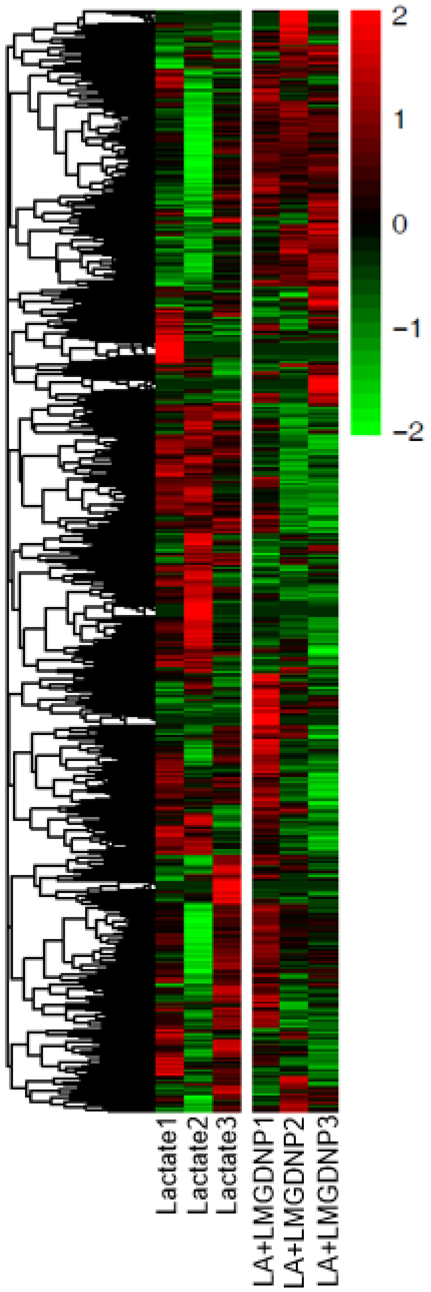

Figure S8. Heatmap with hierarchical clustering of transcriptome gene expressions of NPCs cultured with or without LMGDNP in the presence of lactate. The colors reflect the normalized gene expression of samples. LA, lactate.

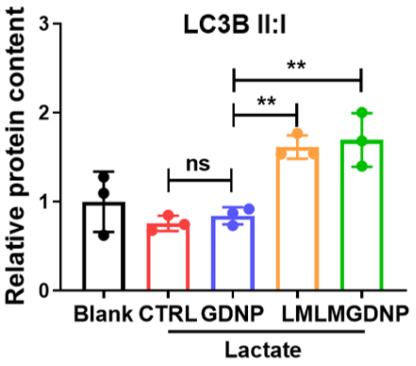

Figure S9. Densitometric analysis of LC3B II: I in NPCs treated with Lactate and GDNP, LM, or LMGDNP for 24h. Data are presented as the mean ± SD, n = 3, *p < 0.05, **p < 0.01 between groups.

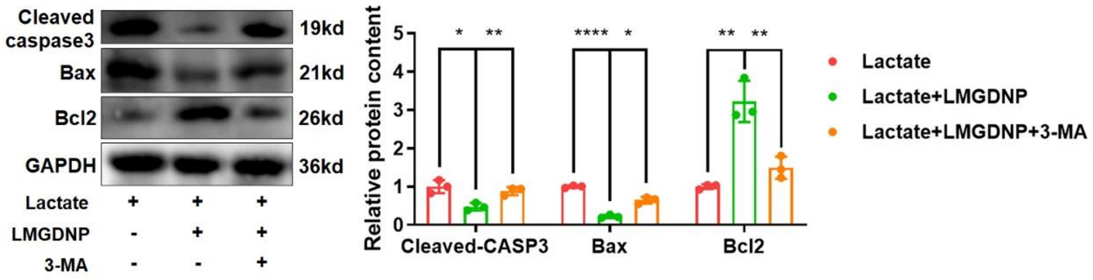

Figure S10. Blots and densitometric analysis of apoptosis-related proteins in NPCs treated with microspheres, lactate, and 3-MA. Data are presented as the mean ± SD, n = 3, *p < 0.05, **p < 0.01, ****p < 0.0001 between groups.

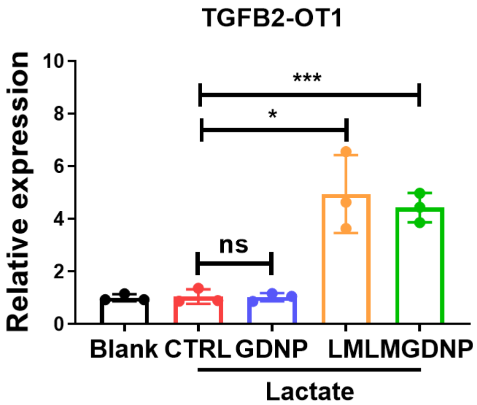

Figure S11. Relative mRNA expression of TGFB2-OT1 in NPCs treated with lactate and GDNP, LM, or LMGDNP. Data are presented as the mean ± SD, n = 3, ns, no significance, *p < 0.05, ***p < 0.001 between groups.

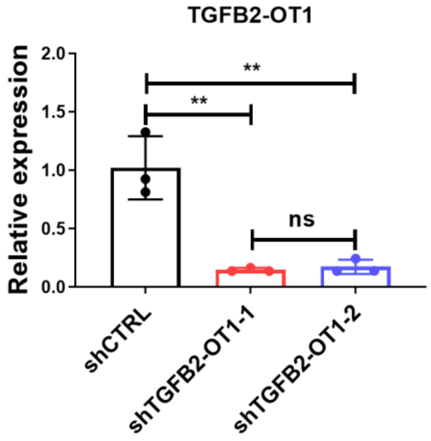

Figure S12. The efficacy of shTGFB2-OT1 on downregulating the expression of TGFB2-OT1. Data are presented as the mean ± SD, n = 3. **p < 0.01 between groups.

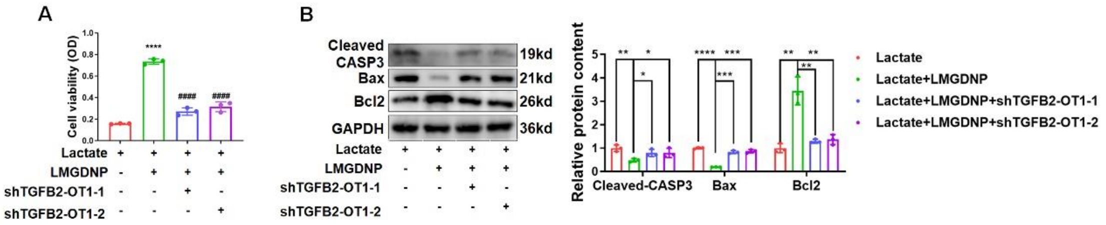

Figure S13. TGFB2-OT1 inhibition impairs cell viability and increases cellular apoptosis. A) The viability of cells transfected with shTGFB2-OT1, which were treated with lactate and LMGDNP for 24h. Results were shown as mean ± SD, n=3, ****p < 0.0001, compared to Lactate; ####p < 0.0001, compared to Lactate + LMGDNP. B) Blots and densitometric analysis of apoptosis-related proteins in NPCs. Data are presented as the mean ± SD, n = 3, *p < 0.05, **p < 0.01, ***p < 0.001, ****p < 0.0001 between groups.

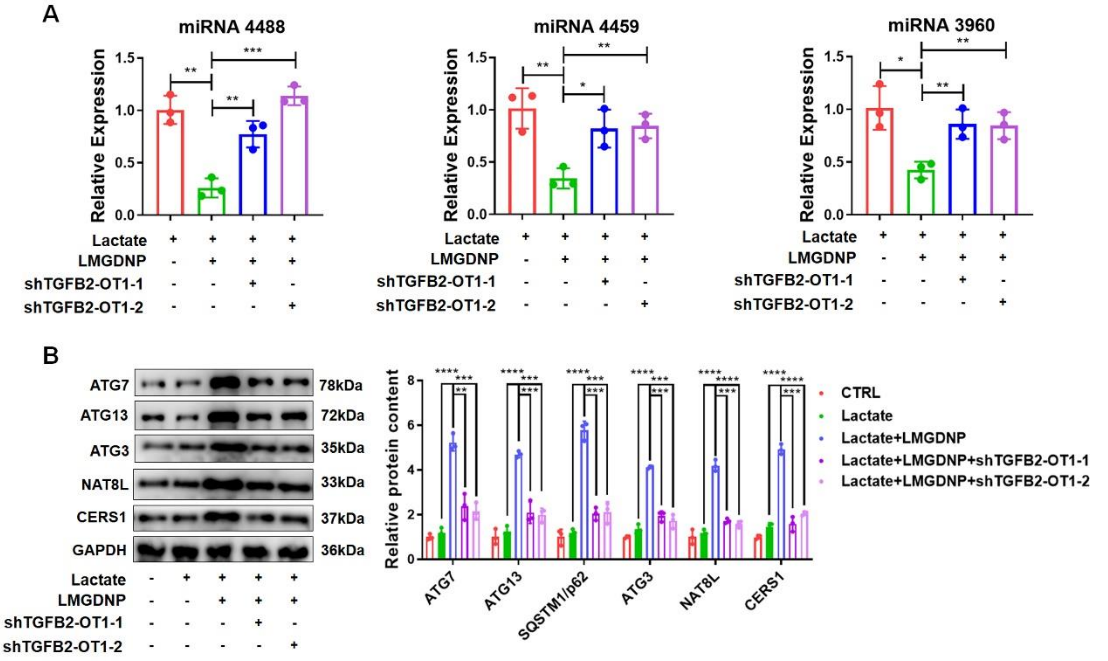

Figure S14. Alteration of TGFB2-OT1 downstream miRNAs and proteins related to autophagy. A) Relative mRNA expression of miRNA 4459, miRNA 3960, and miRNA 4488 in NPCs transfected with shTGFB2-OT1 and exposed to Lactate and LMGDNP for 24h. B) Western blot analysis of autophagy-related proteins (CERS1, NAT8L, ATG13, ATG3 and ATG7). Data are presented as the mean ± SD, n = 3, ns, no significance, *p < 0.05, **p < 0.01, ***p < 0.001 between groups.

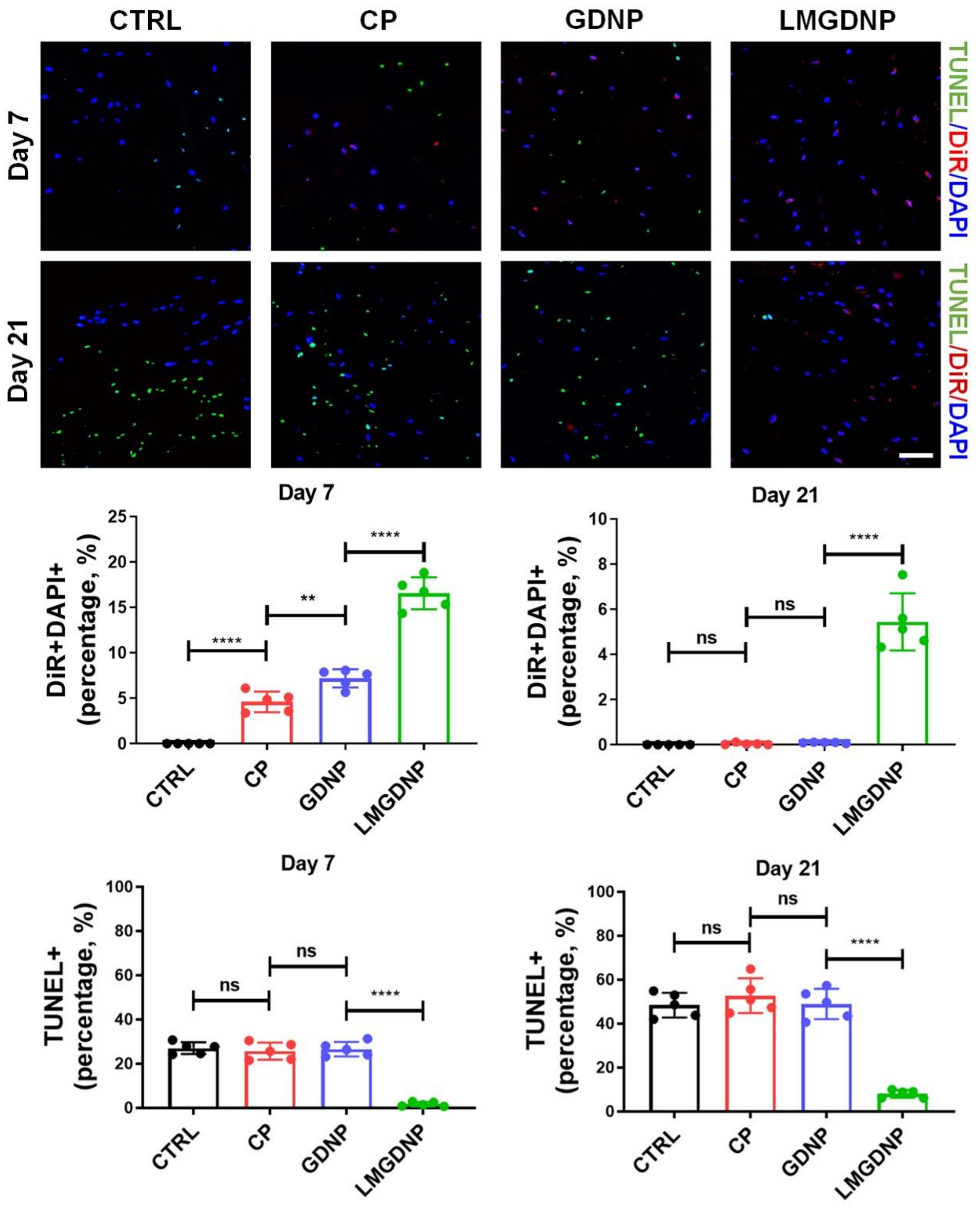

Figure S15. TUNEL staining of histological sections and quantitative analysis of DiR+DAPI+ and TUNEL+ cells in intervertebral discs. Data are presented as the mean ± SD, n=5, ns, no significance, $^{**}p < 0.01$, $^{****}p < 0.0001$ between groups.

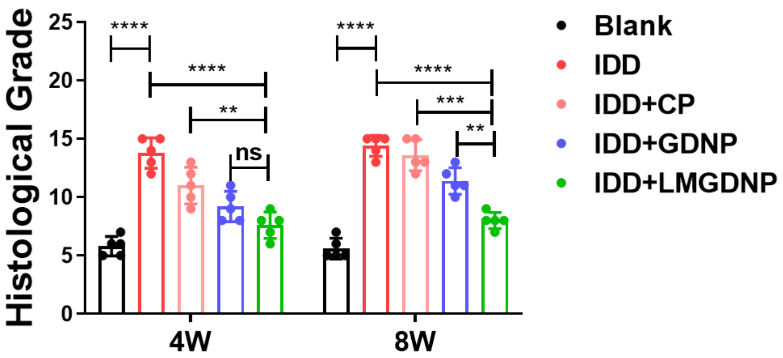

Figure S16. Histological grading scale based on Han et al. Data are presented as the mean ± SD, n=5, ns, no significance, **p < 0.01, ***p < 0.001, ****p < 0.0001 between groups.

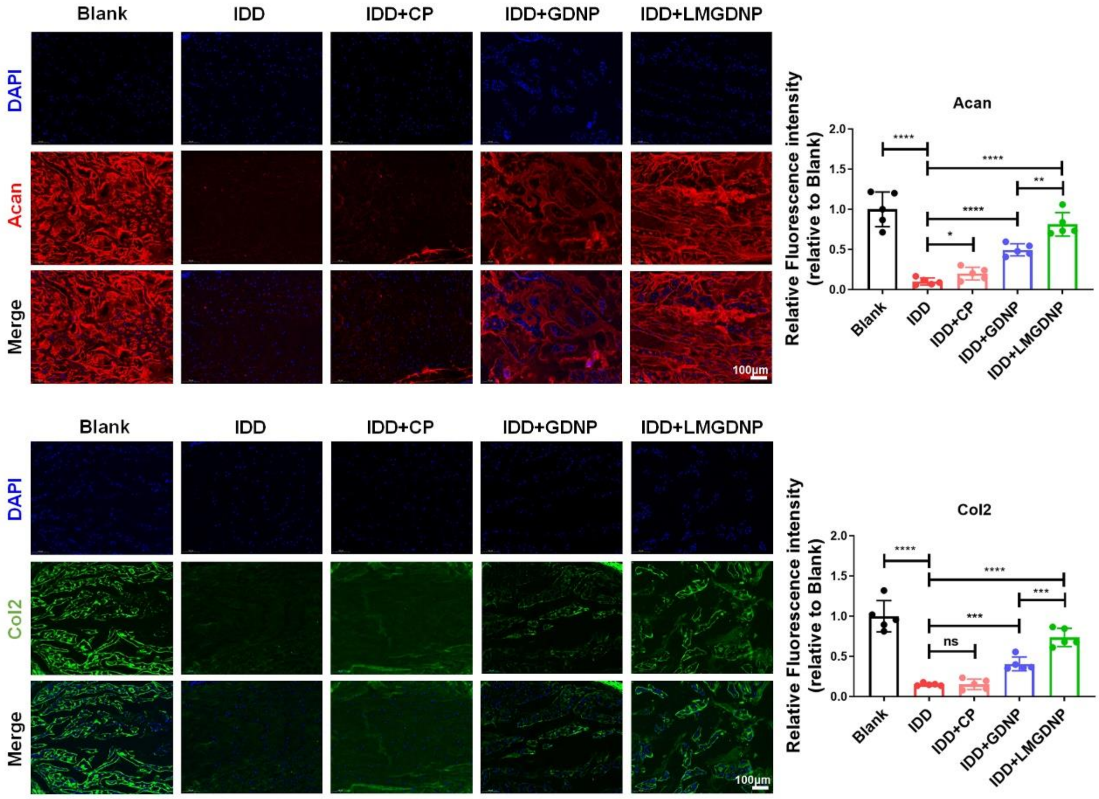

Figure S17. Representative immunofluorescence images of Acan (red) and Col2 (green). Scale bars, 100 μm. Data are presented as the mean ± SD, n = 5, ns, no significance, *p < 0.05, **p < 0.01, ***p < 0.001, ****p < 0.0001 between groups.
